# Supplementary material for: Heterologous Expression and Biochemical Characterization of a New α-Amylase from Nocardiopsis aegyptia HDN19-252 of Antarctic Animal Origin
Source: Mar Drugs. 2025 Apr 4;23(4):159. doi: 10.3390/md23040159 (PMC12028427; doi:10.3390/md23040159)
Supplement: Supplementary file 1 [file marinedrugs-23-00159-s001.zip › marinedrugs-3537184-supplementary.pdf]

Supplementary Material

Heterologous expression and biochemical characterization of a new α-amylase from *Nocardiopsis aegyptia* HDN19-252 of Antarctic animal origin

Fuhao Liu [1,2,†], Xiangnan Zheng [1,3,†], Wenhui Liao [1], Xingtao Ren[1], Chuanteng Ma [1], Guojian Zhang[1,2], Qian Che[1,2], Tianjiao Zhu[1,2], Wenxue Wang [1,2], Tao Zhang [3,*], Feng Han [1,2,*] and Dehai Li [1,2,*]

[1] Key Laboratory of Marine Drugs, Ministry of Education, School of Medicine and Pharmacy, Ocean University of China, 5 Yushan Road, Qingdao 266003, China; 17863905950@163.com (F.L.); zhengxiangnan@qidu-pharma.com (X.D.); liaowenhui726@163.com (W.L.); xingtao.ren@gmail.com (X.R.); ma_chuanteng@163.com (C.M.); zhangguojian@ouc.edu.cn (G.Z.); cheqian064@ouc.edu.cn (Q.C.); zhutj@ouc.edu.cn (T.Z.); bx_wwx@163.com (W.W.).

[2] Laboratory for Marine Drugs and Bioproducts, Qingdao Marine Science and Technology center, Qingdao 266237, China

[3] Shandong Qidu Pharmaceutical Co., Ltd, 17 Hongda Road, Linzi District, Zibo 255400, China

* Correspondence: zhangtao226@163.com (T.Z.); fhan@ouc.edu.cn (F.H.); dehaili@ouc.edu.cn (D.L.); Tel.: +86-532-82031619 (D.L.)

† These authors contributed equally to this work.

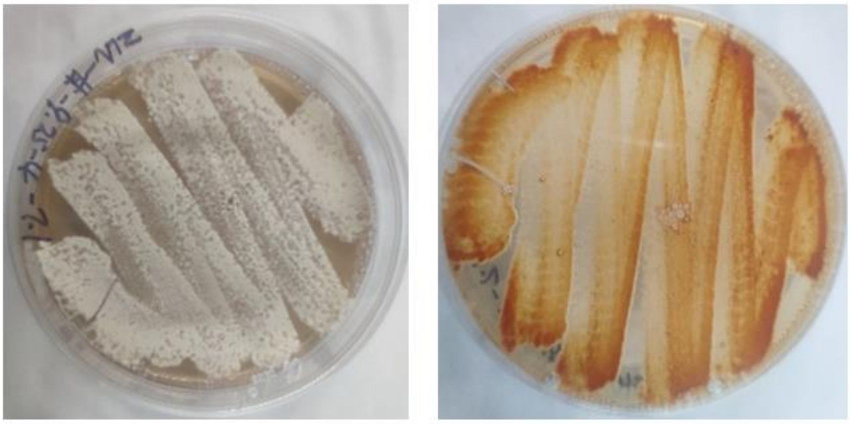

Figure S1. Photographs of the HDN19-252 strain

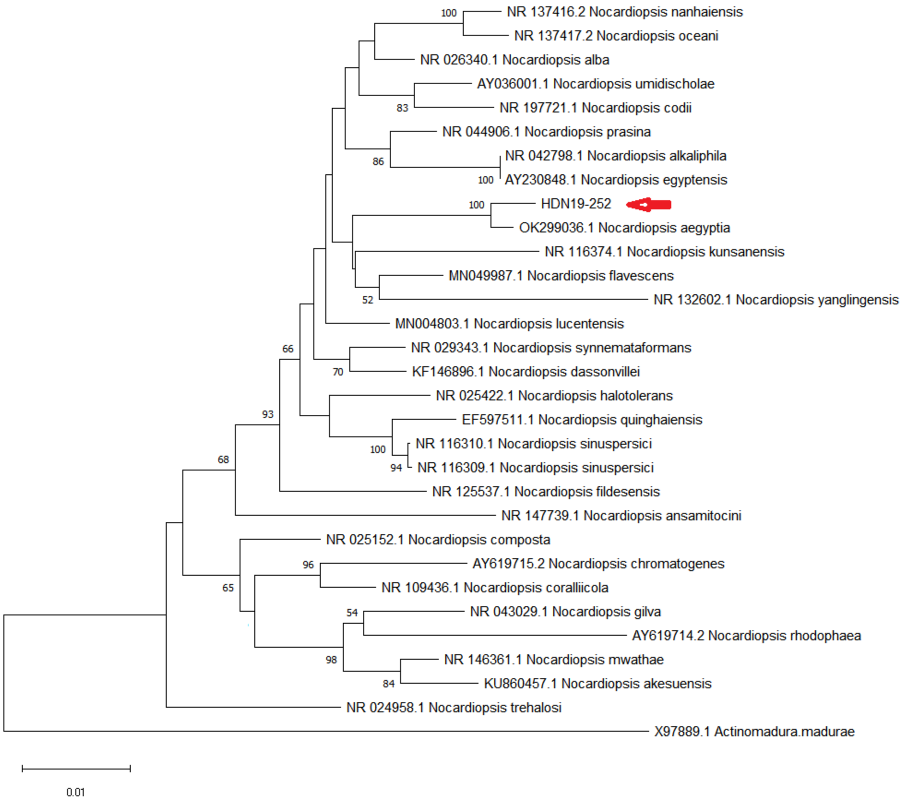

Figure S2. The ITS sequence phylogenetic tree of strain HDN19-252. The ITS sequence phylogenetic tree of strain HDN19-252. Bootstrap values are shown on nodes in percentages of 500 replicates, when greater than 50%. *Actinomadura rhodophaea* was used as an outgroup. GenBank accession numbers are given in parentheses before the genus and species of the bacteria. Bar, 10 substitutions per 1000 nt.

The 16S RNA sequence of HDN19-252：

GGAAGTGCGGCGTGCTACACATGCAGTCGAGCGGTAAGGCCCTTCGGGGTACACGAG
CGGCGAACGGGTGAGTAACACGTGAGCAACCTGCCCCCGACTCCGGGATAAGCGGTG
GAAACGCCGTCTAATACCGGATACGACCGGCCACCTCATGGTAGCCGGTGGAAAGTTT
TTCGGTTGGGGATGGGCTCGCGGCCTATCAGCTTGTTGGTGGGGTAAAGGCCTACCAA

GGCGATTACGGGTAGCCGGCCTGAGAGGGCGACCGGCCACACTGGGACTGAGACACG
GCCCAGACTCCTGCGGGAGGCAGCAGTGGGGAATATTGCGCAATGGGCGAAAGCCTG
ACGCAGCGACGCCGCGTGGGGGATGACGGCCTTCGGGTTGTAAACCTCTTTACCACC
AACGCAGGCTCCCAGTTCTCTGGGGGTTGACGGTAGGTGGGGAATAAGGACCGGCTA
ACTACGTGCCAGCAGCCGCGGTAATACGTAGGGTCCGAGCGTTGTCCGGAATTATTGG
GCGTAAAGAGCTCGTAGGCGGCGTGTCGCGTCTGCTGTGAAAGACCGGGGCTTAACTC
CGGTTCTGCAGTGGATACGGGCATGCTAGAGGTAGGTAGGGGAGACTGGAATTCCTGG
TGTAGCGGTGAAATGCGCAGATATCAGGAGGAACACCGGTGGCGAAGGCGGGTCTCT
GGGCCTTACCTGACGCTGAGGAGCGAAAGCATGGGGAGCGAACAGGATTAGATACCC
TGGTAGTCCATGCCGTAAACGTTGGGCGCTAGGTGTGGGGACTTTCCACGGTTTCCGC
GCCGTAGCTAACGCATTAAGCGCCCCGCCTGGGGAGTACGGCCGCAAGGCTAAAACTC
AAAGGAATTGACGGGGGCCCGCACAAGCGGCGGAGCATGTTGCTTAATTCGACGCAA
CGCGAAGAACCTTACCAAGGTTTGACATCACCCGTGGACCTGCAGAGATGTGGGGTCA
TTTAGTTGGTGGGTGACAGGTGGTGCATGGCTGTCGTCAGCTCGTGTCGTGAGATGTT
GGGTTAAGTCCCGCAACGAGCGCAACCCTTATTCCATGTTGCCAGCACGTAGTGGTGG
GGACTCATGGGAGACTGCCGGGGTCAACTCGGAGGAAGGTGGGGACGACGTCAAGTC
ATCATGCCCCTTATGTCTTGGGCTGCAAACATGCTACAATGGCCGGTACAATGGGCGTG
CGATACCGTAAGGTGGAGCGAATCCCTAAAAGCCGGTCTCAGTTCGGATTGGGGTCTG
CAACTCGACCCCATGAAGGTGGAGTCGCTAGTAATCGCGGATCAGCAACGCCGCGGTG
AATACGTTCCCGGGCCTTGTACACACCGCCCGTCACGTCATGAAAGTCGGCAACACCC
GAAACTTGTGGCCTAACCCTTCGGGGAGGGAATGAGGAAGTTGTCCGACTTT

The 16S RNA sequences of other genera and strains in the phylogenetic tree：

>MK209104 Penicillium sp.
CGGGTCCACCTCCCACCCGTGTCTCTTGAATACCCTGTTGCTTTGGCGGGCCCACCGG
GCCACCCCCGGTCGCCGGGGGGCACCGCGCCCCCGGGCCCGCGCCCGCCAGAGCGCC
TCTGAACCCTAATGAAGAAGGACTGTCTGAGTCTACGATATAATTATCAAAACTTTCAA
CAATGGATCTCTTGGTTCCGGCATCGATGAAGAACGCAGCGAAATGCGATAAGTAATGT
GAATTGCAGAATTCCGTGAATCATCGAATCTTTGAACGCACATTGCGCCCCCTGGCATT
CCGGGGGGCATGCCTGTCCGAGCGTCATTTCTGCCCTCAAGCCCGGCTTGTGTGTTGG
GCGTGGTCCCCCCGGTGTCGGGGGGACCTGCCCCAAAGGCAGCGGCGACGTTCCGCC
TAGGTCCTCGAGCGTATGGGGCTTTGTCACCCGCTCGGGAGGGGCCTACGGGCGTTGG
CCATCCACCAATTTTTTACGGTTGACCTCGGATCAGGTAGGAGTTACCCGCTGAACTTA
AGCATATCAATA

>AY619715.2 Nocardiopsis chromatogenes 16S ribosomal RNA gene, partial sequence
TTACGCGTGCCAGTCCAGCGGTAAGGCCCTTCGGGGTACACGAGCGGCGAACGGGTG
AGTAACACGTGAGCAACCTGCCCCCGACACCGGGATAAGCCGTGGAAACGCGGTCTA
ATACCGGATACGACACGCCCCCGCATGGGGTGCGTGTGGAAAGGTCTTTTCTGGTCGG
GGATGGGCTCGCGGCCTATCAGCTTGTTGGTGGGGTAAAGGCCTACCAAGGCGATTAG
GGTAGCCGGCCTGAGAGGGCGACCGGCCACACTGGGACTGAGACACGGCCCAGACTC
CTGCGGGAGGCAGCAGTGGGGAATCTTGCGCAATGGGCGAAAGCCTGACGCAGCGAC
GCCGCGTGGGGGATGACGGCCTTCGGGTTGTAAACCTCTTTACCACTCACGCAGGCC
CCGGGTTCTCCCGGGGTTGACGGTAGATGGGGAATAAGGACCGGCTAACTACGTGCCA
GCAGCCGCGGTAATACGTAGGGTCCGAGCGTTGTCCGGAATTATTGGGCGTAAAGAGC
TCGTAGGCGGCGTGTCGCGTCTGCTGTGAAAGACCGGGGCTTAACCCCGGTTTTGCAG

TGGATACGGGCATGCTAGAGGTAGGTAGGGGAGACTGGAATTCCTGGTGTAGCGGTGA
AATGCGCAGATATCAGGAGGAACACCGGTGGCGAAGGCGGGTCTCTGGGCCTTACCTG
ACGCTGAGGAGCGAAAGCGTGGGGAGCGAACAGGATTAGATACCCTGGTAGTCCACG
CCGTAAACGTTGGGCGCTAGGTGTGGGGGCTTTCCACGGTTCCCGTGCCGCAGCTAAC
GCATTAAGCGCCCCGCCTGGGGAGTACGGCCGCAAGGCTAAAACTCAAAGGAATTGA
CGGGGGCCCGCACAAGCGGCGGAGCATGTTGCTTAATTCGACGCAACGCGAAGAACC
TTACCAAGGTTTGACATCACCGGTAATCCATCGGAGACGGTGGGTCCTTCGGGGATCG
GTGACAGGTGGTGCATGGCTGTCGTCAGCTCGTGTCGTGAGATGTTGGGTTAAGTCCC
GCAACGAGCGCAACCCTTGTTCCATGTTGCCAGCACGTGATGGTGGGGACTCATGGGA
GACTGCCGGGGTCAACTCGGAGGAAGGTGGGGATGACGTCAAGTCATCATGCCCCTTA
TGTCTTGGGCTGCAAACATGCTACAATGGCCGGTACAGTGGGCGTGCGATGCCGCAAG
GCGGAGCGAATCCCTAAAAGCCGGTCTCAGTTCGGATTGGGGTCTGCAACTCGACCCC
ATGAAGGTGGAGTCGCTAGTAATCGCGGATCAGCAATGCCGCGGTGAATACGTTCCCG
GGCCTTGTACACACCGCCCGTCACGTCATGAAAGTCGGCAACACCCGAAACNTGCGG
CCCAACCCCTTTTGTGGGAGGGAGTGAGTGAAGGTGGGGCTGGCGATTCCGCC
>AY619714.2 Nocardiopsis rhodophaea 16S ribosomal RNA gene, partial sequence
GGGCGTGTTTTAACACATGCAANGTTNGAGCCGTTAAGGCCCCTTTGGGGGGGTACCN
GGANCGGCGAAACGGGGTGAGTAACCACGTGAGCAACCTGCCCCTGATTTTTGGGAT
AAGCCGTGGAAACGCGTTTTAATCCCGGATAGGACACCCCACGCCATGGTGGGGTGTG
GAAAGTTGTGTCGGTTGGGGATGGGCTCGCGGCCTATCAGCTTGTTGGTGGGGTGATG
GCCTACCAAGGCGATTACGGGTAGCCGGCCTGAGAGGGCGACCGGCCACACTGGGAC
TGAGACACGGCCCAGACTCCTGCGGGAGGCAGCAGTGGGGAATATTGCGCAATGGGC
GGAAGCCTGACGCAGCGACGCCGCGTGGGGGATGACGGCCTTCGGGTTGTAAACCTC
TTTTACCACTCACGCAGGCCCCGGGTGTTCTCGGGGGTTGACSGTTAGGTGGGGAATT
AAGGACCGGGCTAACTACGTGCCAGCAGCCGCGGTAATACGTAGGGTCCGAGCGTTGT
CCGGAATTATTGGGCGTAAAGAGCTCGTAGGCGGTGTGTCGCGTCTGCTGTGAAAGGC
TGGGGCTTAACCCTGGTTTTGCAGTGGATACGGGCATGCTAGAGGTAGGTAGGGGAGA
CTGGAATTCCTGGTGTAGCGGTGAAATGCGCAGATATCAGGAGGAACACCGGTGGCGA
AGGCGGGTCTCTGGGCCTTACCTGACGCTGAGGAGCGAAAGCGTGGGGAGCGAACAG
GATTAGATACCCTGGTAGTCCACGCCGTAAACGTTGGGCGCTAGGTGTGGGGGCTTTCC
ACGGTTCCCGTGCCGTAGCTAACGCATTAAGCGCCCCGCCTGGGGAGTACGGCCGCAA
GGCTAAAACTCAAAGGAATTTGACGGGGGCCCGCACAAGCGGCGGAGCATGTTGCTT
AATTCGACGCAACGCGAAGAACCTTACCAAGGTTTGACATCNCCGGTAATCCATTAGA
GACAGNGGGTCCTTTTGGGATCGGTGACAGGTGGTGCATGGCTGTCGTCAGCTCGTGT
CGTGAGATGTTGGGTTAAGTCCCGCAACGAGCGCAACCCTTGTTCCATGTTGCCAGCA
CGTGGTGGTGGGGACTCATGGGAGACTGCCGGGGTCAACTCGGAGGAAGGTGGGGAT
GACGTCAAGTCATCATGCCCCTTATGTCTTGGGCTGCAAACATGCTACAATGGCCGGTA
CAGTGGGCGTGCGATGCCGTAAGGTGGAGCGAATCCCTAAAAGCCGGTCTCAGTTCGG
ATTGGGGTCTGCAACTCGACCCCATGAAGGTGGAGTCGCTAGTAATCGCGGATCAGCA
GTGCCGCGGTGAATACGTTCCCGGGCCTTGTACACACCGCCCGTCACGTCATGAAAGT
CGGCAACACCCGAAACCTGTGGCCTAACCCTTCGGGGAGGGAGTGGGTGAAGGTGGG
GCTGGCGATTGGGACGAAGTCGTAACAAGGTAGCCGTACCGGAAGG
>NR_137416.2 Nocardiopsis nanhaiensis strain 10A08B 16S ribosomal RNA, partial sequence
TTCAGAGTTTGATCCTGGCTCAGGACGAACGCTGGCGGCGTGCTTAACACATGCAAGT

CGAGCGGTAAGGCCCTTCGGGGTACACGAGCGGCGAACGGGTGAGTAACACGTGAGC
AACCTGCCCCTGACTCTGGGATAAGCGGTGGAAACGCCGTCTAATACCGGATACGACA
CACCGTCTCATGGCGTGTGTGTGGAAAGTTTTTCGGTCAGGGATGGGCTCGCGGCCT
ATCAGCTTGTTGGTGGGGTAAAGGCCTACCAAGGCGATTACGGGTAGCCGGCCTGAGA
GGGCGACCGGCCACACTGGGACTGAGACACGGCCCAGACTCCTACGGGAGGCAGCA
GTGGGGAATATTGCACAATGGGCGAAAGCCTGATGCAGCGACGCCGCGTGGGGGATG
ACGGCCTTCGGGTTGTAAACCTCTTTTACCATCAACGCAGGCTCCGGGTTCTCCCGGG
GTTGACGGTAGGTGGGGAATAAGGACCGGCTAACTACGTGCCAGCAGCCGCGGTAATA
CGTAGGGTCCGAGCGTTGTCCGGAATTATTGGGCGTAAAGAGCTCGTAGGCGGCGTGT
CGCGTCTGCTGTGAAAGACCGGGGCTTAACTCCGGTTTGGCAGTGGATACGGGCACGC
TAGAGGTAGGTAGGGGAGACTGGAATTCCTGGTGTAGCGGTGAAATGCGCAGATATCA
GGAGGAACACCGGTGGCGAAGGCGGGTCTCTGGGCCTTACCTGACGCTGAGGAGCGA
AAGCATGGGGAGCGAACAGGATTAGATACCCTGGTAGTCCATGCCGTAAACGTTGGGC
GCTAGGTGTGGGGACTTTCCACGGTTTCCGCGCCGTAGCTAACGCATTAAGCGCCCCG
CCTGGGGAGTACGGCCGCAAGGCTAAAACTCAAAGGAATTGACGGGGGCCCGCACAA
GCGGCGGAGCATGTTGCTTAATTCGACGCAACGCGAAGAACCTTACCAAGGTTTGACA
TCACCCGTGGACCTGTAGAGATACAGGGTCATTTAGTTGGTGGGTGACAGGTGGTGCA
TGGCTGTCGTCAGCTCGTGTCGTGAGATGTTGGGTTAAGTCCCGCAACGAGCGCAACC
CTTATTCCATGTTGCCAGCACGTAGTGGTGGGGACTCATGGGAGACTGCCGGGGTCAA
CTCGGAGGAAGGTGGGGACGACGTCAAGTCATCATGCCCCTTATGTCTTGGGCTGCAA
ACATGCTACAATGGCCGGTACAATGGGCGTGCGATACCGTAAGGTGGAGCGAATCCCTT
AAAGCCGGTCTCAGTTCGGATTGGGGTCTGCAACTCGACCCCATGAAGGTGGAGTCGC
TAGTAATCGCGGATCAGCAACGCCGCGGTGAGTACGTTCCCGGGCCTTGTACACACCG
CCCGTCACGTCATGAAAGTCGGCAACACCCGAAACTTGTGGCCTAACCCCTTGTGGGA
GGGAATGAGTGAAGGTGGGGCTGGCGATTGGGACGAAGTCGTAACAAGGTAGCCGTA
CCGGAAGGTGCGGCTGGATCACCTCCTAA
>NR_137417.2 Nocardiopsis oceani strain 10A08A 16S ribosomal RNA, complete sequence
GCTACTTCGAGTTTGATCCTGGCTCAGGACGAACGCTGGCGGCGTGCTTAACACATGC
AAGTCGAGCGGTAAGGCCCTTCGGGGCACACGAGCGGCGAACGGGTGAGTAACACGT
GAGCAACCTGCCCCTGACTCTGGGATAAGCGGCGGAAACGCCGTCTAATACCGGATAC
GACGCACCGTCTCATGGCGTGTGTGTGGAAAGTTTTTCGGTCAGGGATGGGCTCGCG
GCCTATCAGCTTGTTGGTGGGGTAAAGGCCTACCAAGGCGATTACGGGTAGCCGGCCT
GAGAGGGCGACCGGCCACACTGGGACTGAGACACGGCCCAGACTCCTACGGGAGGC
AGCAGTGGGGAATATTGCACAATGGGCGAAAGCCTGATGCAGCGACGCCGCGTGGGG
GATGACGGCCTTCGGGTTGTAAACCTCTTTTACCACCAACGCAGGCTCCGGGTTCTCTC
GGGGTTGACGGTAGGTGGGGAATAAGGACCGGCTAACTACGTGCCAGCAGCCGCGGT
AATACGTAGGGTCCGAGCGTTGTCCGGAATTATTGGGCGTAAAGAGCTCGTAGGCGGC
GCGTCGCGTCTGCTGTGAAAGACCGGGGCTTAACTCCGGTTTGGCAGTGGATACGGGC
ACGCTAGAGGTAGGTAGGGGAGACTGGAATTCCTGGTGTAGCGGTGAAATGCGCAGAT
ATCAGGAGGAACACCGGTGGCGAAGGCGGGTCTCTGGGCCTTACCTGACGCTGAGGA
GCGAAAGCATGGGGAGCGAACAGGATTAGATACCCTGGTAGTCCATGCCGTAAACGTT
GGGCGCTAGGTGTGGGGACTTTCCACGGTTTCCGCGCCGTAGCTAACGCATTAAGCGC
CCCGCCTGGGGAGTACGGCCGCAAGGCTAAAACTCAAAGGAATTGACGGGGGCCCGC
ACAAGCGGCGGAGCATGTTGCTTAATTCGACGCAACGCGAAGAACCTTACCAAGGTTT

GACATCACCCGTGGACCTGTAGAGATACAGGGTCATTTAGTTGGTGGGTGACAGGTGG
TGCATGGCTGTCGTCAGCTCGTGTCGTGAGATGTTGGGTTAAGTCCCGCAACGAGCGC
AACCCTTATTCCACGTTGCCAGCACGTAATGGTGGGGACTCATGGGAGACTGCCGGGG
TCAACTCGGAGGAAGGTGGGGACGACGTCAAGTCATCATGCCCCTTATGTCTTGGGCT
GCAAACATGCTACAATGGCCGGTACAATGGGCGTGCGATACCGTAAGGTGGAGCGAAT
CCCTTAAAGCCGGTCTCAGTTCGGATTGGGGTCTGCAACTCGACCCCATGAAGGTGGA
GTCGCTAGTAATCGCGGATCAGCAACGCCGCGGTGAATACGTTCCCGGGCCTTGTACA
CACCGCCCGTCACGTCATGAAAGTCGGCAACACCCGAAACTTGTGGCCTAACCCCTTG
TGGGAGGGAATGAGTGAAGGTGGGGCTGGCGATTGGGACGAAGTCGTAACAAGGTAG
CCGTACCGGAAGGTGCGGCTGGATCACCTCCTAA
>NR_147739.1 Nocardiopsis ansamitocini strain EGI 80425 16S ribosomal RNA, partial sequence
CAGAGTTTGATCCTGGCTCAGGACGAACGCTGGCGGCGTGCTTAACACATGCAAGTCG
AGCGGTAAGGCCCTTCGGGGTACACGAGCGGCGAACGGGTGAGTAACACGTGAGTAA
CCTGCCCCCAACTCTGGGATAAGCCGAGGAAACTCGGTCTAATACCGGATATGACACG
CTCCTGCATGGGAGGGTGTGGAAAGTTTTTCGGTTGGGGATGGACTCGCGGCCTATC
AGCTTGTTGGTGAGGTAATGGCTCACCAAGGCGATAACGGGTAGCCGGCCTGAGAGG
GCGACCGGCCACACTGGGACTGAGACACGGCCCAGACTCCTGCGGGAGGCAGCAGT
GGGGAATATTGCGCAATGGGCGAAAGCCTGACGCAGCGACGCCGCGTGGGGGATGAC
GGCCTTCGGGTTGTAAACCTCTTTACCACTCACGCAGGCTCCCAGTTTTCTGGGGGTT
GACGGTAGGTGGGGAATAAGGACCGGCTAACTACGTGCCAGCAGCCGCGGTAATACGT
AGGGTCCGAGCGTTGTCCGGAATTATTGGGCGTAAAGAGCTCGTAGGCGGCTAGTCGC
GTCTGCTGTGAAAGACCGGGGCTTAACTCCGGTTCTGCAGTGGATACGGGCTGGCTAG
AGGTAGGTAGGGGAGACTGGAATTCCTGGTGTAGCGGTGAAATGCGCAGATATCAGGA
GGAACACCGGTGGCGAAGGCGGGTCTCTGGGCCTTACCTGACGCTGAGGAGCGAAAG
CATGGGGAGCGAACAGGATTAGATACCCTGGTAGTCCATGCCGTAAACGTTGGGCGCT
AGGTGTGGGGACTTTCCACGGTCTCCGTGCCGTAGCTAACGCATTAAGCGCCCCGCCT
GGGGAGTACGGCCGCAAGGCTAAACTCAAAGGAATTGACGGGGGCCCGCACAAGCG
GCGGAGCATGTTGCTTAATTCGACGCAACGCGAAGAACCTTACCAAGGTTTGACATCA
CCCATGGACTCGTGGAGACACGGGGTCATTTAGTTGGTGGGTGACAGGTGGTGCATGG
CTGTCGTCAGCTCGTGTCGTGAGATGTTGGGTTAAGTCCCGCAACGAGCGCAACCCTT
GTTCCATGTTGCCAGCACGTAATGGTGGGGACTCATGGGAGACTGCCGGGGTCAACTC
GGAGGAAGGTGGGGATGACGTCAAGTCATCATGCCCCTTATGTCTTGGGCTGCAAACA
TGCTACAATGGCCGGTACAGAGGGCGTGCGATGTCGTAAGGCGGAGCGAATCCCTAAA
AGCCGGTCTCAGTTCGGATTGGGGTCTGCAACTCGACCCCATGAAGGTGGAGTCGCTA
GTAATCGCGGATCAGCAACGCCGCGGTGAATACGTTCCCGGGCCTTGTACACACCGCC
CGTCACGTCACGAAAGTCGGCAACACCCGAAACTTGCGGCCCAACCCTTCGGGGAGG
GAGTGAGTGAAGGTGGGGCTGGCGATTGGGACGAAGTCGTAACAAGGTAGCCGTACC
GGAAGGTGCGGCTGGATCACCTCCT
>NR_043029.1 Nocardiopsis gilva YIM 90087 16S ribosomal RNA, partial sequence
GAACGCTGGCGGCGTGCTTAACACATGCAAGTCGAGCGGTAAGGCCCCTTCGGGGGT
ACACGAGCGGCGAACGGGTGAGTAACACGTGAGCAACCTGCCCCTGACTCTGGGATA
AGCCGTGGAAACGCGGTCTAATACCGGATACGACACCCTGCCGCATGGCGGGGTGTGG
AAAGTACTTGTCGGTTGGGGATGGGCTCGCGGCCTATCAGCTTGTTGGTGGGGTGAAG

GCCTACCAAGGCGATTACGGGTAGCCGGCCTGAGAGGGCGACCGGCCACACTGGGAC
TGAGACACGGCCCAGACTCCTGCGGGAGGCAGCAGTGGGGAATCTTGCGCAATGGGC
GAAAGCCTGACGCAGCGACGCCGCGTGGGGGATGACGGCCTTCGGGTTGTAAACCTC
TTTTACCACTCACGCAGGCCCCGGGTTTTCTTGGGGTTGACGGTAGGTGGGGAATAAG
GACCGGCTAACTACGTGCCAGCAGCCGCGGTAATACGTAGGGTCCGAGCGTTGTCCGG
AATTATTGGGCGTAAAGAGCTCGTAGGCGGTGTGTCGCGTCTGCTGTGAAAGGCTGGG
GCTTAACCCTGGTTTTGCAGTGGATACGGGCATGCTAGAGGTAGGTAGGGGAGACTGG
AATTCCTGGTGTAGCGGTGAAATGCGCAGATATCAGGAGGAACACCGGTGGCGAAGG
CGGGTCTCTGGGCCTTACCTGACGCTGAGGAGCGAAAGCGTGGGGAGCGAACAGGAT
TAGATACCCTGGTAGTCCACGCCGTAAACGTTGGGCGCTAGGTGTGGGGGCTTTCCAC
GGTTCCCGTGCCGTAGCTAACGCATTAAGCGCCCCGCCTGGGGAGTACGGCCGCAAGG
CTAAAACTCAAAGGAATTGACGGGGGCCCGCACAAGCGGCGGAGCATGTTGCTTAATT
CGACGCAACGCGAAGAACCTTACCAAGGTTTGACATCACCGGTAAACCATCGGAGAC
GGTGGGTCCTTTTGGGATCGGTGACAGGTGGTGCATGGCTGTCGTCAGCTCGTGTCGT
GAGATGTTGGGTTAAGTCCCGCAACGAGCGCAACCCTTGTTCCATGTTGCCAGCACGT
GATGGTGGGGACTCATGGAGACTGCCGGGGTCAACTCGGAGGAAGGTGGGGATGAC
GTCAAGTCATCATGCCCCTTATGTCTTGGGCTGCAAACATGCTACAATGGCCGGTACAG
TGGGCGTGCGATGCCGTGAGGTGGAGCGAATCCCTAAAAGCCGGTCTCAGTTCGGATT
GGGGTCTGCAACTCGACCCCATGAAGGTGGAGTCGCTAGTAATCGCGGATCAGCAGTG
CCGCGGTGAATACGTTCCCGGGCCTTGTACACACCGCCCGTCACGTCATGAAAGTCGG
CAACACCCGAAACCTGCGGCCTAACCAGCTTGCTGGGGGGAGTGGGTGAAGGTGGGG
CTGGCGATTGGGACGAAGTCGTAACAAGGTAGCCGTACCGGAAGG
>AY036001.1 Nocardiopsis umidischolae 16S ribosomal RNA gene, partial sequence
GGCGTGCTTAACACATGCAAGTCGAGCGGTAAGGCCCTTCGGGGTACACGAGCGGCG
AACGGGTGAGTAACACGTGAGCAACCTGCCCCTGACTCTGGGATAAGCGGTGGAAAC
GCCGTCTAATACCGGATACGACACACGGCCTCATGGCGGTGTGTGGAAAGTTTTTCGG
TCAGGGATGGGCTCGCGGCCTATCAGCTTGTTGGTGGGGTAACGGCCTACCAAGGCGA
TTACGGGTAGCCGGCCTGAGAGGGCGACCGGCCACACTGGGACTGAGACACGGCCCA
GACTCCTGCGGGAGGCAGCAGTGGGGAATATTGCGCAATGGGCGAAAGCCTGACGCA
GCGACGCCGCGTGGGGGATGACGGCCTTCGGGTTGTAAACCTCTTTTACCACCAACGC
AGGCTCCCAGTTCTCTGGGGGTTGACGGTAGGTGGGGAATAAGGACCGGCTAACTACG
TGCCAGCAGCCGCGGTAATACGTAGGGTCCGAGCGTTGTCCGGAATTATTGGGCGTAA
AGAGCTCGTAGGCGGCGTGTCACGTCTGCTGTGAAAGACCGGGGCTTAACTCCGGTTC
TGCAGTGGATACGGGCATGCTAGAGGTAGGTAGGGGAGACTGGAATTCCTGGTGTAGC
GGTGAAATGCGCAGATATCAGGAGGAACACCGGTGGCGAAGGCGGGTCTCTGGGCCT
TACCTGACGCTGAGGAGCGAAAGCATGGGGAGCGAACAGGATTAGATACCCTGGTAGT
CCATGCCGTAAACGTTGGGCGCTAGGTGTGGGGACTTTCCACGGTTTCCGCGCCGCAG
CTAACGCATTAAGCGCCCCGCCTGGGGAGTACGGCCGCAAGGCTAAAACTCAAAGGA
ATTGACGGGGGCCCGCACAAGCGGCGGAGCATGTTGCTTAATTCGACGCAACGCGAA
GAACCTTACCAAGGTTTGACATCGCCCGTGGACCTGTAGAGATACAGGGTCATTTAGTT
GGTGGGTGACAGGTGGTGCATGGCTGTCGTCAGCTCGTGTCGTGAGATGTTGGGTTAA
GTCCCGCAACGAGCGCAACCCCTATTCTATGTTGCCAGCACGTTATGGTGGGGACTCAT
AGGAGACTGCCGGGGTCAACTCGGAGGAAGGTGGGGACGACGTCAAGTCATCATGCC
CCTTATGTCTTGGGCTGCAAACATGCTACAATGGCCGGTACAATGGGCGTGCGAGACC

GCAAGGTGGAGCGAATCCCTAAAAGCCGGTCTCAGTTCGGATTGGGGTCTGCAACTCG
ACCCCATGAAGGTGGAGTCGCTAGTAATCGCGGATCAGCAACGCCGCGGTGAATACGT
TCCCGGGCCTTGTACACACCGCCCGTCACGTCATGAAAGTCGGCAACACCCGAAACTT
GTGGCCTAACCCTTCGGGGAGGGAATGAGTGAAGGTGGGGCTGGCGATTGGGACGAA
GTCGTAACAA

>EF597511.1 Nocardiopsis quinghaiensis strain YIM 28A4 16S ribosomal RNA gene, partial sequence

TTTGAGTTTGATCCTGGCTCAGGACGAACGCTGGCGGCGTGCTTAACACATGCAAGTC
GAGCGGTAAGGCCCTTCGGGGTACACGAGCGGCGAACGGGTGAGTAACACGTGAGCA
ACCTGCCCCCGACTCCGGGATAAGC
GGTGGAAACGCCGTCTAATACCGGATACGACCCATGGTCTCCTGGCCGTGGGTGGAAA
GTTTCTCGGTTGGGGATGGGCTCGCGGCCTATCAGCTAGTTGGTGGGGTAACAGCCTA
CCAAGGCGATTACGGGTAGCCGGCCTGAGAGGGCGACCGGCCACACTGGGACTGAGA
CACGGCCCAGACTCCTACGGGAGGCAGCAGTGGGGAATATTGCGCAATGGGCGAAAG
CCTGACGCAGCGACGCCGCGTGGGGGATGACGGCCTTCGGGTTGTAAACCTCTTTTAC
CACCAACGCAGGCTCCCAGTGCTCTGGGGGTTGACGGTAGGTGGGGAATAAGGACCG
GCTAACTACGTGCCAGCAGCCGCGGTAATACGTAGGGTCCGAGCGTTGTCCGGAATTAT
TGGGCGTAAAGAGCTCGTAGGCGGCGTGTCGCGTCTGCTGTGAAAGACCGGGGCTTA
ACCCCGGTTCTGCAGTGGATACGGGCATGCTAGAGGTAGGTAGGGGAGACTGGAATTC
CTGGTGTAGCGGTGAAATGCGCAGATATCAGGAGGAACACCGGTGGCGAAGGCGGGT
CTCTGGGCCTTACCTGACGCTGAGGAGCGAAAGCATGGGGAGCGAACAGGATTAGATA
CCCTGGTAGTCCATGCCGTAAACGTTGGGCGCTAGGTGTGGGGACTTTCCACGGTTTC
CGCGCCGTAGCTAACGCATTAAGCGCCCCGCCTGGGGAGTACGGCCGCAAGGCTAAAA
CTCAAAGGAATTGACGGGGGCCCGCACAAGCGGCGGAGACATGTTGCTTAATTCGAC
GCAACGCGAAGAACCTTACCAAGGTGGGACATCACCCGTGGACCTGCAGAGATGTGG
GGTCATTTAGTTGGCGGGTGACAGGTGGTGCATGGCTGTCGTCAGCTCGTGTCGTGAG
ATGTTGGGTTAAGTCCCGCAACGGCAGCGCAACCCTTATTCCATGTTGCCAGCACGTG
ATGGTGGGGACTCATGGGAGACTGCCGGGGTCAACTCGGAGGAAGGTGGGGATGACG
TCAAGTCATCATGCCCCTTATGTCTTGGGCTGCAAACATGCTACAATGGCCGGTACAAT
GGGCGTGCGATGCCGCAAGGTGGAGCGAATCCCTAAAAGCCGGTCTCAGTTCGGATTG
GGGTCTGCAACTCGACCCCATGAAGGTGGAGTCGCTAGTAATCGCGGATCAGCAACGC
CGCGGTGAATACGTTCCCGGGCCTTGCACACA

>NR_197721.1 Nocardiopsis codii strain CT-R113 16S ribosomal RNA, partial sequence

TGCAAGTCGAGCGGTAAGGCCCTTCGGGGTACACGAGCGGCGAACGGGTGAGTAACA
CGTGAGCAACCTGCCCCTGACTCCGGGATAAGCGGTGGAAACGCCGTCTAATACCGGA
TATGACACGCCACCTCATGGTGGTGTGTGGAAAGTTTTTCGGTCAGGGATGGGCTCGC
GGCCTATCAGCTTGTTGGTGGGGTAACGGCCTACCAAGGCGATTACGGGTAGCCGGCC
TGAGAGGGCGACCGGCCACACTGGGACTGAGACACGGCCCAGACTCCTGCGGGAGG
CAGCAGTGGGGAATATTGCGCAATGGGCGAAAGCCTGACGCAGCGACGCCGCGTGGG
GGATGACGGCCTTCGGGTTGTAAACCTCTTTTACCACCAACGCAGGCTCCCAGTTCTCT
GGGGGTTGACGGTAGGTGGGGAATAAGGACCGGCTAACTACGTGCCAGCAGCCGCGG
TAATACGTAGGGTCCGAGCGTTGTCCGGAATTATTGGGCGTAAAGAGCTCGTAGGCGG
CGTGTCGCGGTCTGCTGTGAAAAGACCCGGGGGCTTAACTCCGGTTCTGCAGTGGATA
CGGGCATGGCTAGAGGTAGGTAGGGGAAAACTGGAATTCCTGGTGTAGCGGTGAAA

ATGCGCAGATATCAGGAGGAACACCGGTGGCGAAGGCGGGTTTCTGGGGCCTTTACCT
GACGCTGAGGAAGCGAAAGCATGGGGAGCGAACAGGATTAGATACCCTGGTAGTCCA
TGCCGTAAACGTTGGGCGCTAGGTGTGGGGACTTTCCACGGTTTCCGCGCCGCAGCTA
ACGCATTAAGCGCCCCGCCTGGGGAGTACGGCCGCAAGGCTAAAACTCAAAGGAATT
GACGGGGGCCCGCACAAGCGGCGGAGCATGTTGCTTAATTCGACGCAACGCGAAGAA
CCTTACCAAGGTTTGACATCGCCCGTGGACCTGTAGAGATACAGGGTCATTTAGTTGGT
GGGTGACAGGTGGTGCATGGCTGTCGTCAGCTCGTGTCGTGAGATGTTGGGGTTAAGT
CCCGCAACGAGCGCAACCCCTTTCTATGTTGCCAGCACGTAATGGTGGGGACTCATAG
GAGACTGCCGGGGTCAACTCGGAGGAAGGTGGGGAGGACGTCAAGTCATCATGCCCC
TTATGTCTTGGGCTGCAAACATGCTACAATGGCCGGTACAATGGGCGTGCGAGACCGT
AAGGTGGAGCGAATCCCTAAAAGCCGGTCTCAGTTCGGATTGGGGTCTGCAACTCGAC
CCC

>NR_116374.1 Nocardiopsis kunsanensis strain JSM 073097 16S ribosomal RNA, partial sequence

CACGAGCGGCGAACGGGTGAGTAACACGTGAGCAACCTGCCCCTGACTCCGGGATAA
GCGGTGGAAACGCCGTCTAATACCGGATACGACTCTCCGGCTCATGCTGGAGGGTGGA
AAGTTTTTTCGGTCGGGGATGGGCTCGCGGCCTATCAGCTTGTTGGTGGGGTAATGGCC
CACCAAGGCGATTACGGGTAGCCGGCCTGAGAGGGTGACCGGCCACACTGGGACTGA
GACACGGCCCAGACTCCTGCGGGAGGCAGCAGTGGGAAATCTTGCGCAATGGGCGAA
AGCCTGACGCAGCGACGCCGCGTGGGGGATGACGGCCTTCGGGTGTAAACTCCTTTTA
CCACTCACGCAGGCTCCCAGTCTCTGGGGGTTGACGGTAAGTGGGGAATAAGGACCG
GCTAACTACGTGCCAGCAGCCGCGGTAATACGTAGGGTCCGAGCGTTGTCCGGAATTAT
TGGGCGTAAAGAGCTCGTAGGCGGCATGTCACGTCTGCTGTGAAAGACCGGGGCTTAA
CTTCGGTTTTGCAGTGGATACGGGCATGCTAGAGGTAGGTAGGGGAGACTGGAATTCC
TGGTGTAGCGGTGGAATGCGCAGATATCAGGAGGAACACCGGTGGCGAAGGCGGGTC
TCTGGGCCTTACCTGACGCTGAGGAGCGAAAGCATGGGTAGCGAACAGGATTAGATAC
CCTGGTAGTCCATGCCGTAAACGATGGGCGCTAGGTGTGGGGACTTTCCACGGTTTCC
GCGCCGTAGCTAACGCATTAAGCGCCCCGCCTGGGGAGTACGGCCGCAAGGCTAAAAC
TCAAAGGAATTGACGGGGGCCCGCACAAGCGGCGGAGCATGTTGCTTAATTCGACGC
AACGCGAAGAACCTTACCAAGGTTTGACATCACCCGTGGACCTGCAGAGATGTGGGG
TCATTTAGTTGGTGGGTGACAGGTGGTGCATGGCTGTCGTCAGCTCGTGTCGTGAGA
TGTTGGGTTAAGTCCCGTAACGAGCGCAACCCTTGTTCCATGTTGCCAGCACGTAGTG
GTGGGGACTCATGGGAGACTGCCGGGGTCAACTCGGAGGAAGGTGGGGACGACGTC
AAGTCATCATGCCCCTTATGTCTTGGGCTGCAAACATGCTACAATGGCCGGTACAATGG
GCGTGCGAGACCGTAAGGTGGAGCGAATCCCTAAAAGCCGGTCTCAGTTCGGATTGG
GGTCTGCAACTCGACCCCATGAAGGTGGAGTCGCTAGTAATCGCGGATCAGCAACGCC
GCGGTGAATACGTTCCCGGGCCTTGTACACACCGCCCGTCACGTCATGAAAGTCGGCA
ACACCCGAAACTTGCGGCCCAACCCCGTGTGGGAGGGAGTGAGTGAAGGTGGGGCTG
GCGATTGGGACGAAGTCGTAA

>NR_116310.1 Nocardiopsis sinuspersici strain HM7 16S ribosomal RNA, partial sequence

TTTGATCCTGGCTCAGGACGAACGCTGGCGGCGTGCTTAACACATGCAAGTCGAGCGG
TAAGGCCCTTCGGGGTACACGAGCGGCGAACGGGTGAGTAACACGTGAGCAACCTGC
CCCTGACTCCGGGATAAGCGGTGGAAACGCCGTCTAATACCGGATACGACCCATGGTC
TCCTGACCGTGGGTGGAAAGTTTTTCGGTCGGGGATGGGCTCGCGGCCTATCAGCTTG

TTGGTGGGGTAACAGCCTACCAAGGCGATTACGGGTAGCCGGCCTGAGAGGGCGACC
GGCCACACTGGGACTGAGACACGGCCCAGACTCCTACGGGAGGCAGCAGTGGGGAAT
ATTGCGCAATGGGCGAAAGCCTGACGCAGCGACGCCGCGTGGGGGATGACGGCCTTC
GGGTTGTAAACCTCTTTACCACCAACGCAGGCTCCCAGTTCTCTGGGGGTTGACGGT
AGGTGGGGAATAAGGACCGGCTAACTACGTGCCAGCAGCCGCGGTAATACGTAGGGTC
CGAGCGTTGTCCGGAATTATTGGGCGTAAAGAGCTCGTAGGCGGCGTGTCGCGTCTGC
TGTGAAAGACCGGGGCTTAACCCCGGTTCTGCAGTGGATACGGGCATGCTAGAGGTAG
GTAGGGGAGACTGGAATTCCTGGTGTAGCGGTGAAATGCGCAGATATCAGGAGGAACA
CCGGTGGCGAAGGCGGGTCTCTGGGCCTTACCTGACGCTGAGGAGCGAAAGCATGGG
GAGCGAACAGGATTAGATACCCTGGTAGTCCATGCCGTAAACGTTGGGCGCTAGGTGT
GGGGACTTTCCACGGTTTCCGCGCCGTAGCTAACGCATTAAGCGCCCCGCCTGGGGAG
TACGGCCGCAAGGCTAAAACTCAAAGGAATTGACGGGGGCCCGCACAAGCGGCGGAG
CATGTTGCTTAATTCGACGCAACGCGAAGAACCTTACCAAGGTTTGACATCACCCGTG
GACCTGCAGAGATGTGGGGTCATTTAGTTGGCGGGTGACAGGTGGTGCATGGCTGTCG
TCAGCTCGTGTCGTGAGATGTTGGGTTAAGTCCCGCAACGAGCGCAACCCTTATTCCAT
GTTGCCAGCACGTAATGGTGGGGACTCATGGGAGACTGCCGGGGTCAACTCGGAGGA
AGGTGGGGATGACGTCAAGTCATCATGCCCCTTATGTCTTGGGCTGCAAACATGCTACA
ATGGCCGGTACAATGGGCGTGCGATGCCGCAAGGTGGAGCGAATCCCTAAAAGCCGGT
CTCAGTTCGGATTGGGGTCTGCAACTCGACCCCATGAAGGTGGAGTCGCTAGTAATCG
CGGATCAGCAACGCCGCGGTGAATACGTTCCCGGGCCTTGTACACACCGCCCGTCACG
TCATGAAAGTCGGCAACACCCGAAACTTGCGGCCTAACCCTTCGGGGAGGGAGTGAG
TGAAGGTGGGGCTGGCGATTGGGACGAAGTCGTAACAAGGTAGCCGTACCGGAAGGT
GCGGCTGGATCACCTCCTT
>NR_146361.1 Nocardiopsis mwathae strain No.156 16S ribosomal RNA, partial sequence
TCAGGACGAACGCTGGCGGCGTGCTTAACACATGCAAGTCGAGCGGTAAGGCCCTTTC
GGGGGTACACGAGCGGCGAACGGGTGAGTAACACGTGAGTAACCTGCCCCTGACTCC
GGGATAAGCCGTGGAAACGCGGTCTAATACCGGATACGACGCCCCTCCGCATGGTGGG
GTGTGGAAAGTTGTTTCGGTTGGGGATGGACTCGCGGCCTATCAGCTTGTTGGTGGGG
TAACGGCCTACCAAGGCGATTACGGGTAGCCGGCCTGAGAGGGCGACCGGCCACACT
GGGACTGAGACACGGCCCAGACTCCTGCGGGAGGCAGCAGTGGGGAATATTGCGCAA
TGGGCGGAAGCCTGACGCAGCGACGCCGCGTGGGGGATGACGGCCTTCGGGTTGTAA
ACCTCTTTACCACTCACGCAGGCCCGGGGTTTTCTTCGGGTTGACGGTAGGTGGGGA
ATAAGGACCGGCTAACTACGTGCCAGCAGCCGCGGTAATACGTAGGGTCCGAGCGTTG
TCCGGAATTATTGGGCGTAAAGAGCTCGTAGGCGGTGTGTCGCGTCTGCTGTGAAAGG
CTGGGGCTCAACCCTGGTTTTGCAGTGGATACGGGCATGCTAGAGGTAGGTAGGGGAG
ACTGGAATTCCTGGTGTAGCGGTGAAATGCGCAGATATCAGGAGGAACACCGGTGGCG
AAGGCGGGTCTCTGGGCCTTACCTGACGCTGAGGAGCGAAAGCGTGGGGAGCGAACA
GGATTAGATACCCTGGTAGTCCACGCCGTAAACGTTGGGCGCTAGGTGTGGGGGCTTT
CCACGGTTCCCGTGCCGTAGCTAACGCATTAAGCGCCCCGCCTGGGGAGTACGGCCGC
AAGGCTAAAACTCAAAGGAATTGACGGGGGCCCGCACAAGCGGCGGAGCATGTTGCT
TAATTCGACGCAACGCGAAGAACCTTACCAAGGTTTGACATCACCGGTAATCCGTTAG
AGATAGCGGGTCCTTCGGGGATCGGTGACAGGTGGTGCATGGCTGTCGTCAGCTCGTG
TCGTGAGATGTTGGGTTAAGTCCCGCAACGAGCGCAACCCTTGTTCCATGTTGCCAGC
ACGTAGTGGTGGGGACTCATGGGAGACTGCCGGGGTCAACTCGGAGGAAGGTGGGGA

TGACGTCAAGTCATCATGCCCCTTATGTCTTGGGCTGCAAACATGCTACAATGGCCGGT
ACAGTGGGCGTGCGATGCCGTGAGGCGGAGCGAATCCCTAAAAGCCGGTCTCAGTTC
GGATTGGGGTCTGCAACTCGACCCCATGAAGGTGGAGTCGCTAGTAATCGCGGATCAG
CATTGCCGCGGTGAATACGTTCCCGGGCCTTGTACACACCGCCCGTCACGTCATGAAA
GTCGGCAACACCCGAAACCTGTGGCCTAACCCCTTGTGGGAGGGAGTGGGTGAAGGT
GGGGCTGGCGATTGGGACGAAG
>NR_132602.1 Nocardiopsis yanglingensis strain A18 16S ribosomal RNA, partial sequence
AATAACCATGTCGCTAGATTACCTGCAGTCGAGCGGTAAGGCCCTTCGGGGTACACGA
GCGGCGAACGGGTGAGTAACACGTGAGCAACCTGCCCCTGACTCCGGGATAAGCGGT
GGAAACGCCGTCTAATACCGGATACGACCCTCCTCCGCATGGTGGGGGGTGGAAAGTT
TTTTCGGTCGGGGATGGGCTCGCGGCCTATCAGCTTGTTGGTGGGGTAACGGCCTACC
AAGGCGATTACGGGTAGCCGGCCTGAGAGGGCGACCGGCCACACTGGGACTGAGACA
CGGCCCAGACTCCTGCGGGAGGCAGCAGTGGGGAATATTGCGCAATGGGCGAAAGCC
TGACGCAGCGACGCCGCGTGGGGGATGACGGCCTTCGGGTTGTAAACCTCTTTTACCA
CTCACGCAGGCCCCACGTTTTCGTGGGGTTGACGGTAGGTGGGGAATAAGGACCGGCT
AACTACGTGCCAGCAGCCGCGGTAATACGTAGGGTCCGAGCGTTGTCCGGAATTATTG
GGCGTAAAGAGCTCGTAGGCGGCGTGTCGCGTCTGCTGTGAAAGACCGGGGCTTAAC
CCCGGTTCTGCAGTGGATACGGGCATGCTAGAGGTAGGTAGGGGAGACTGGATTTCCT
GGTGTAGCGGTGAAATGCGCAGATATCAGGAGGAACACCGGTGGCGAAGGCGGGTCT
CTGGGCCTTACCTGACGCTGAGGAGCGAAAGCATGGGGAGCGAACAGGATTAGATAC
CCTGGTAGTCCATGCCGTAAACGTTGGGCGCTAGGTGTGGGGACTTTCCACGGTTTCC
GCGCCGTAGCTAACGCATTAAGCGCCCCGCCTGGGGAGTACGGCCGCAAGGCTAAAAC
TCAAAGGAATTGACGGGGGCCCGCACAAGCGGCGGAGCATGTTGCTTTATTTCGACGC
AACGCGAAGAACCTTACCAAGGTTTGACATCACCCGTGGACCTGTAGAGATACAGGGT
CATTTGGTTGGCGGGTGACAGTTGTTGCATGGCTGTCGTCAGCTCGTGTCGTGCGATGT
TGGGTTAAGTCCCGCAACTAGCGCAACCCTTGTTCCATGTTGTCAGCACGTTATTGTGG
GGACTCATTGGAGAACTGCCGGGGTCAACTCGGAGGAAGGTGGGGACGACGTCAAGT
CATCATGCCCCTTATGTCTTGGGCTGCAAACATGCTACAATGGCCGGTACAATGGGCGT
GCGATACCGTGAGGTGGAGCGAATCCCTAAAAGCCGGTCTCAGTTCGGATTGGGGTCT
GCAACTCGACCCCATGAAGGTGGAGTCGCTAGTAATCGCGGATCAGCAACGCCGCGGT
GAATACGTTCCCGGGCCTTGTACACACCGCCCGTCACGTCATGAAAGTCGGCAACACC
CGAAACTTGTGGCCTAACCCTTCGGGGAGGGAGTGAGTGAAGGTGGGGCTGGCGATG
GACGAAGTCGAACAAGAGCCACTTGCG
>NR_125537.1 Nocardiopsis fildesensis strain GW9-2 16S ribosomal RNA, partial sequence
GACGAACGCTGGCGGCGTGCTTAACACATGCAAGTCGAGCGGTAAGGCCCCTTCGGG
GGTACACGAGCGGCGAACGGGTGAGTAACACGTGAGCAACCTGCCCCTGACTCCGGG
ATAAGCGGTGGAAACGCCGTCTAATACCGGATACGACCCGCGGCCTCATGGCGGCGGG
TGGAAAGTTCTTCGGTCGGGGATGGGCTCGCGGCCTATCAGCTTGTTGGTGGGGTAAC
GGCCTACCAAGGCGATTACGGGTAGCCGGCCTGAGAGGGCGACCGGCCACACTGGGA
CTGAGACACGGCCCAGACTCCTGCGGGAGGCAGCAGTGGGGAATATTGCGCAATGGG
CGGAAGCCTGACGCAGCGACGCCGCGTGGGGGATGACGGCCTTCGGGTTGTAAACCT
CTTTTACCACCGACGTAGGCTCCGGGTGTTCTCGGGGTTGACGGTAGGTGGGGAATAA
GGACCGGCTAACTACGTGCCAGCAGCCGCGGTAATACGTAGGGTCCGAGCGTTGTCCG
GAATTATTGGGCGTAAAGAGCTCGTAGGCGGCGTGTCGCGTCTGCTGTGAAAGACCGG

GGCTTAACTCCGGTTCTGCAGTGGATACGGGCATGCTAGAGGTAGGTAGGGGAGACTG
GAATTCCTGGTGTAGCGGTGAAATGCGCAGATATCAGGAGGAACACCGGTGGCGAAG
GCGGGTCTCTGGGCCTTACCTGACGCTGAGGAGCGAAAGCATGGGGAGCGAACAGG
ATTAGATACCCTGGTAGTCCATGCCGTAAACGTTGGGCGCTAGGTGTGGGGGCTTTCCA
CGGTCTCCGCGCCGTAGCTAACGCATTAAGCGCCCCGCCTGGGGAGTACGGCCGCAAG
GCTAAAACTCAAAGGAATTGACGGGGGCCCGCACAAGCGGCGGAGCATGTTGCTTAA
TTCGACGCAACGCGAAGAACCTTACCAAGGTTTGACATCACCCGTGGACCCGCAGAG
ATGCGGGGTCATTTGGTTGGCGGGTGACAGGTGGTGCATGGCTGTCGTCAGCTCGTGT
CGTGAGATGTTGGGTTAAGTCCCGCAACGAGCGCAACCCTTGTTCCATGTTGCCAGCA
CGTGATGGTGGGGACTCATGGGAGACTGCCGGGGTCAACTCGGAGGAAGGTGGGGAC
GACGTCAAGTCATCATGCCCCTTATGTCTTGGGCTGCAAACATGCTACAATGGCCGGTA
CAGTGGGCATGCGATGCCGTGAGGCGGAGCGAATCCCTTAAAGCCGGTCTCAGTTCGG
ATTGGGGTCTGCAACTCGACCCCATGAAGGTGGAGTCGCTAGTGATCGCGGATCAGCA
ACGCCGCGGTGAATACGTTCCCGGGCCTTGTACACACCGCCCGTCACGTCATGAAAGT
CGGCAACACCCGAAACTTGTGGCCCAACCCCTTGTGGGAGGGAGTGAGTGAAGGTGG
GGCTGGCGATTGGGACG
>NR_116309.1 Nocardiopsis sinuspersici strain HM6 16S ribosomal RNA, partial sequence
TTTGATCCTGGCTCAGGACGAACGCTGGCGGCGTGCTTAACACATGCAAGTCGAGCGG
TAAGGCCCTTCGGGGTACACGAGCGGCGAACGGGTGAGTAACACGTGAGCAACCTGC
CCCTGACTCCGGGATAAGCGGTGGAAACGCCGTCTAATACCGGATACGACCCATGGTC
TCCTGACCGTGGGTGGAAAGTTTTTCGGTCGGGGATGGGCTCGCGGCCTATCAGCTTG
TTGGTGGGGTAACAGCCTACCAAGGCGATTACGGGTAGCCGGCCTGAGAGGGCGACC
GGCCACACTGGGACTGAGACACGGCCCAGACTCCTACGGGAGGCAGCAGTGGGGAAT
ATTGCGCAATGGGCGAAAGCCTGACGCAGCGACGCCGCGTGGGGGATGACGGCCTTC
GGGTTGTAAACCTCTTTACCACCAACGCAGGCTCCCGGTTCTCTGGGGGTTGACGGT
AGGTGGGGAATAAGGACCGGCTAACTACGTGCCAGCAGCCGCGGTAATACGTAGGGTC
CGAGCGTTGTCCGGAATTATTGGGCGTAAAGAGCTCGTAGGCGGCGTGTCGCGTCTGC
TGTGAAAGACCGGGGCTTAACCCCGGTTCTGCAGTGGATACGGGCATGCTAGAGGTAG
GTAGGGGAGACTGGAATTCCTGGTGTAGCGGTGAAATGCGCAGATATCAGGAGGAACA
CCGGTGGCGAAGGCGGGTCTCTGGGCCTTACCTGACGCTGAGGAGCGAAAGCATGGG
GAGCGAACAGGATTAGATACCCTGGTAGTCCATGCCGTAAACGTTGGGCGCTAGGTGT
GGGGACTTTCCACGGTTTCCGCGCCGTAGCTAACGCATTAAGCGCCCCGCCTGGGGAG
TACGGCCGCAAGGCTAAAACTCAAAGGAATTGACGGGGGCCCGCACAAGCGGCGGAG
CATGTTGCTTAATTCGACGCAACGCGAAGAACCTTACCAAGGTTTGACATCACCCGTG
GACCTGCAGAGATGTGGGGTCATTTAGTTGGCGGGTGACAGGTGGTGCATGGCTGTCG
TCAGCTCGTGTCGTGAGATGTTGGGTTAAGTCCCGCAACGAGCGCAACCCTTATTCCAT
GTTGCCAGCACGTAATGGTGGGGACTCATGGGAGACTGCCGGGGTCAACTCGGAGGA
AGGTGGGGATGACGTCAAGTCATCATGCCCCTTATGTCTTGGGCTGCAAACATGCTACA
ATGGCCGGTACAATGGGCGTGCGATGCCGCAAGGTGGAGCGAATCCCTAAAAGCCGGT
CTCAGTTCGGATTGGGGTCTGCAACTCGACCCCATGAAGGTGGAGTCGCTAGTAATCG
CGGATCAGCAACGCCGCGGTGAATACGTTCCCGGGCCTTGTACACACCGCCCGTCACG
TCATGAAAGTCGGCAACACCCGAAACTTGCGGCCTAACCCTTCGGGGAGGGAGTGAG
TGAAGGTGGGGCTGGCGATTGGGACGAAGTCGTAACAAGGTAGCCGTACCGGAAGGT
GCGGCTGGATCACCTCCTT

>NR_042798.1 Nocardiopsis alkaliphila 16S ribosomal RNA, partial sequence
CRATDBYDNACTBDATMBDNASTNCTTTTATCTTGGCTCAGGACGAACGCTGGCGGCG
TGCTTAACACATGCAAGTCGAGCGGTAAGCCCTTCGGGGTACACGAGCGGCGAACGG
GTGAGTAACACGTGAGCAACCTGCCCCCGACTCCGGGATAAGCGGTGGAAACGCCGT
CTAATACCGGATACGACCCGTCACCTCATGGTGTGCGGGTGGAAAGTTTTTTCGGTTGG
GGATGGGCTCGCGGCCTATCAGCTTGTTGGTGGGGTAACGGCCCACCAAGGCGATTAC
GGGTAGCCGGCCTGAGAGGGCGACCGGCCACACTGGGACTGAGACACGGCCCAGACT
CCTACGGGAGGCAGCAGTGGGGAATATTGCACAATGGGCGCAAGCCTGATGCAGCGA
CGCCGCGTGGGGGATGACGGCCTTCGGGTTGTAAACCTCTTTTACCACTCACGCAGGC
TCCACGTTCTCGTGGGGTTGACGGTCAGTGGGGAATAAGGACCGGCTAACTACGTGCC
AGCAGCCGCGGTAATACGTAGGGTCCGAGCGTTGTCCGGAATTATTGGGCGTAAAGAG
CTCGTAGGCGGCATGTCGCGTCTGCTGTGAAAGACCGGGGCTTAACTCCGGTTCTGCA
GTGGATACGGGCATGCTAGAGGTAGGTAGGGGAGACTGGAATTCCTGGTGTAGCGGTG
AAATGCGCAGATATCAGGAGGAACACCGGTGGCGAAGGCGGGTCTCTGGGCCTTACCT
GACGCTGAGGAGCGAAAGCATGGGTAGCGAACAGGATTAGATACCCTGGTAGTCCATG
CCGTAAACGTTGGGCGCTAGGTGTGGGGACTTTCCACGGTTTCCGCGCCGTAGCTAAC
GCATTAAGCGCCCCGCCTGGGGAGTACGGCCGCAAGGCTAAAACTCAAAGGAATTGA
CGGGGGCCCGCACAAGCGGCGGAGCATGTTGCTTAATTCGACGCAACGCGAAGAACC
TTACCAAGGTTTGACATCACCCGTGGACCTGTAGAGATACAGGGTCATTTGGTTGGCG
GGTGACAGGTGGTGCATGGCTGTCGTCAGCTCGTGTCGTGAGATGTTGGGTTAAGTCC
CGCAACGAGCGCAACCCTTGTTCCATGTTGCCAGCACGTAGTGGTGGGGACTCATGGG
AGACTGCCGGGGTCAACTCGGAGGAAGGTGGGGACGACGTCAAGTCATCATGCCCCT
TATGTCTTGGGCTGCAAACATGCTACAATGGCCGGTACAATGGGCGTGCGATACCGTGA
GGTGGAGCGAATCCCTGAAAGCCGGTCTCAGTTCGGATTGGGGTCTGCAACTCGACCC
CATGAAGGTGGAGTCGCTAGTAATCGCGGATCAGCAACGCCGCGGTGAATACGTTCCC
GGGCCTTGTACACACCGCCCGTCACGTCATGAAAGTCGGCAACACCCGAAACTTGTGG
CCTAACCCTTCGGGGAGGGAATGAGTGAAGGTGGGGCTGGCGATTGGGACGAAGTCG
TAACAAGGTAGCCG
>NR_109436.1 Nocardiopsis coralliicola strain SCSIO 10427 16S ribosomal RNA, partial sequence
GGCCCTTCGGGGTACACGAGCGGCGAACGGGTGAGTAACACGTGAGCAACCTGCCCC
TGACTCTGGGATAAGCGGTGGAAACGCCGTCTAATACCGGATACGACCCGCCCCTGCC
TGGGGTGCGGGTGGAAAGTTTTTCGGTTGGGGATGGGCTCGCGGCCTATCAGCTTGT
TGGTGGGGTAACGGCCTACCAAGGCGATTACGGGTAGCCGGCCTGAGAGGGCGACCG
GCCACACTGGGACTGAGACACGGCCCAGACTCCTGCGGGAGGCAGCAGTGGGGAATC
TTGCGCAATGGGCGAAAGCCTGACGCAGCGACGCCGCGTGGGGGATGACGGCCTTCG
GGTTGTAAACCTCTTTTACCACTCACGCAGGCTCCGGGTTCTCCCGGGGTTGACGGTA
GGTGGGGAATAAGGACCGGCTAACTACGTGCCAGCAGCCGCGGTAATACGTAGGGTCC
GAGCGTTGTCCGGAATTATTGGGCGTAAAGAGCTCGTAGGCGGTGTGTCGCGTCTGCT
GTGAAAGACCGGGGCTTAACTCCGGTTTTGCAGTGGATACGGGCATGCTAGAGGTAGG
TAGGGGAGACTGGAATTCCTGGTGTAGCGGTGAAATGCGCAGATATCAGGAGGAACAC
CGGTGGCGAAGGCGGGTCTCTGGGCCTTACCTGACGCTGAGGAGCGAAAGCGTGGGG
AGCGAACAGGATTAGATACCCTGGTAGTCCACGCCGTAAACGTTGGGCGCTAGGTGTG
GGGGCTTTCCACGGTTCCCGTGCCGCAGCTAACGCATTAAGCGCCCCGCCTGGGGAGT

ACGGCCGCAAGGCTAAAACTCAAAGGAATTGACGGGGGCCCGCACAAGCGGCGGAG
CATGTTGCTTAATTCGACGCAACGCGAAGAACCTTACCAAGGTTTGACATCACCGGTA
AGCCATCGGAGACGGTGGGTCCTTCGGGGATCGGTGACAGGTGGTGCATGGCTGTCGT
CAGCTCGTGTCGTGAGATGTTGGGTTAAGTCCCGCAACGAGCGCAACCCTTGTTCCAT
GTTGCCAGCACGTGATGGTGGGGACTCATGGGAGACTGCCGGGGTCAACTCGGAGGA
AGGTGGGGATGACGTCAAGTCATCATGCCCCTTATGTCTTGGGCTGCAAACATGCTACA
ATGGCCGGTACAGTGGGCGTGCGATACCGCAAGGTGGAGCGAATCCCTAAAAGCCGGT
CTCAGTTCGGATTGGGGTCTGCAACTCGACCCCATGAAGGTGGAGTCGCTAGTAATCG
CGGATCAGCAATGCCGCGGTGAATACGTTCCCGGGCCTTGTACACACCGCCCGTCACG
TCATGAAAGTCGGCAACACCCGAAACTTGCGGCCTAACCCTTGTGGGGGGAGTGAGT
GAAGGTGGGGCTGGCGA
>NR_044906.1 Nocardiopsis prasina DSM 43845 16S ribosomal RNA, partial sequence
CGCTGGCGGCGTGCTTAACACATGCAAGTCGAGCGGTAAGGCCCTTCGGGGTACACGA
GCGGCGAACGGGTGAGTAACACGTGAGCAACCTGCCCCTGACTCCGGGATAAGCGGT
GGAAACGCCGTCTAATACCGGATACGACCCGCCACCTCATGGTGGAGGGTGGAAAGTT
TTATCGGTCAGGGATGGGCTCGCGGCCTATCAGCTTGTTGGTGGGGTAACGGCCTACCA
AGGCGATTACGGGTAGCCGGCCTGAGAGGGCGACCGGCCACACTGGGACTGAGACAC
GGCCCAGACTCCTGCGGGAGGCAGCAGTGGGGAATATTGCACAATGGGCGAAAGCCT
GATGCAGCGACGCCGCGTGGGGGATGACGGCCTTCGGGTTGTAAACCTCTTTTACCAC
CAACGCAGGCTCCACGTTCTCGTGGGGTTGACGGTAGGTGGGGAATAAGGACCGGCT
AACTACGTGCCAGCAGCCGCGGTAATACGTAGGGTCCGAGCGTTGTCCGGAATTATTG
GGCGTAAAGAGCTCGTAGGCGGCATGTCGCGTCTGCTGTGAAAGACCGGGGCTTAACT
CCGGTTCTGCAGTGGATACGGGCATGCTAGAGGTAGGTAGGGGAAACTGGAATTCCTG
GTGTAGCGGTGAAATGCGCAGATATCAGGAGGAACACCGGTGGCGAAGGCGGGTTTC
TGGGCCTTACCTGACGCTGAGGAGCGAAAGCATGGGGAGCGAACAGGATTAGATACC
CTGGTAGTCCATGCCGTAAACGTTGGGCGCTAGGTGTGGGGACTTTCCACGGTTTCCG
CGCCGTAGCTAACGCATTAAGCGCCCCGCCTGGGGAGTACGGCCGCAAGGCTAAAACT
CAAAGGAATTGACGGGGGCCCGCACAAGCGGCGGAGCATGTTGCTTAATTCGACGCA
ACGCGAAGAACCTTACCAAGGTTTGACATCACCCGTGGACCTGTAGAGATACAGGGTC
ATTTAGTTGGTGGGTGACAGGTGGTGCATGGCTGTCGTCAGCTCGTGTCGTGAGATGTT
GGGTTAAGTCCCGCAACGAGCGCAACCCTTGTTCCATGTTGCCAGCACGTAATGGTGG
GGACTCATGGGAGACTGCCGGGGTCAACTCGGAGGAAGGTGGGGACGACGTCAAGTC
ATCATGCCCCTTATGTCTTGGGCTGCAAACATGCTACAATGGCCGGTACAATGGGCGTG
CGATACCGTAAGGTGGAGCGAATCCCTTAAAGCCGGTCTCAGTTCGGATTGGGGTCTG
CAACTCGACCCCATGAAGGTGGAGTCGCTAGTAATCGCGGATCAGCAACGCCGCGGTG
AATACGTTCCCGGGCCTTGTACACACCGCCCGTCACGTCATGAAAGTCGGCAACACCC
GAAACTTGTGGCCTAACCCTTCGGGGAGGGAATGAGTGAAGGTGGGGCTGGCGATTG
GGACGAAGTCGTAACAA
>NR_025152.1 Nocardiopsis composta strain KS9 16S ribosomal RNA, partial sequence
GGCGTGCTTAACACATGCAAGTCGAGCGGTAAGGCCCTTCGGGGGTACACGAGCGGC
GAACGGGTGAGTAACACGTGAGCAACCTGCCCCTGACTCCGGGATAAGCGGTGGAAA
CGCCGTCTAATACCGGATACGACCCTCCGCCTCATGGCGGCGGGTGGAAAGTTTTTCG
GTCGGGGATGGGCTCGCGGCCTATCAGCTTGTTGGTGGGGTAACGGCCTACCAAGGCG
ATTACGGGTAGCCGGCCTGAGAGGGCGACCGGCCACACTGGGACTGAGACACGGCCC

AGACTCCTGCGGGAGGCAGCAGTGGGGAATCTTGCGCAATGGGCGGAAGCCTGACGC
AGCGACGCCGCGTGGGGGATGACGGCCTTCGGGTTGTAAACCTCTTTACCACTCACG
CAGGCCCCGGGTTTTCTCGGGGTTGACGGTAGGTGGGGAATAAGGACCGGCTAACTAC
GTGCCAGCAGCCGCGGTAATACGTAGGGTCCGAGCGTTGTCCGGAATTATTGGGCGTA
AAGAGCTCGTAGGCGGCGTGTCGCGTCTGCTGTGAAAGGCCGGGGCTTAACTCCGGTT
TTGCAGTGGATACGGGCATGCTAGAGGTAGGTAGGGGAGACTGGAATTCCTGGTGTAG
CGGTGAAATGCGCAGATATCAGGAGGAACACCGGTGGCGAAGGCGGGTCTCTGGGCC
TTACCTGACGCTGAGGAGCGAAAGCGTGGGGAGCGAACAGGATTAGATACCCTGGTA
GTCCACGCCGTAAACGTTGGGCGCTAGGTGTGGGGGCTTTCCACGGTTCCCGTGCCGC
AGCTAACGCATTAAGCGCCCCGCCTGGGGAGTACGGCCGCAAGGCTAAAACTCAAAG
GAATTGACGGGGGCCCGCACAAGCGGCGGAGCATGTTGCTTAATTCGACGCAACGCG
AAGAACCTTACCAAGGTTTGACATCACCGGTAATCCTGCAGAGATGTGGGGTCCTTCG
GGGATCGGTGACAGGTGGTGCATGGCTGTCGTCAGCTCGTGTCGTGAGATGTTGGGTT
AAGTCCCGCAACGAGCGCAACCCTTGTTCCATGTTGCCAGCACGTGATGGTGGGGACT
CATGGGAGACTGCCGGGGTCAACTCGGAGGAAGGTGGGGATGACGTCAAGTCATCAT
GCCCCTTATGTCTTGGGCTGCAAACATGCTACAATGGCCGGTACAGTGGGCGTGCGATG
CCGTGAGGCGGAGCGAATCCCTAAAAGCCGGTCTCAGTTCGGATTGGGGTCTGCAACT
CGACCCCATGAAGGTGGAGTCGCTAGTAATCGCGGATCAGCAATGCCGCGGTGAATAC
GTTCCCGGGCCTTGTACACACCGCCCGTCACGTCATGAAAGTCGGCAACACCCGAAAC
CTGTGGCCCAACCCCTTGTGGGAGGGAGTGGGTGAAGGTGGGGCTGGCGATTGGGAC
GAAGTCGTAACAA
>NR_025422.1 Nocardiopsis halotolerans DSM 44410 16S ribosomal RNA, partial sequence
CCTGGCTCAGGACGAACGCTGGCGGCGTGCTTAACACATGCAAGTCGAGCGGTAAGG
CCCTTCGGGGTACACGAGCGGCGAACGGGTGAGTAACACGTGAGCAACCTGCCCCTG
ACTCCGGGATAAGCGGTGGAAACGCCGTCTAATACCGGATACGACCCTTGGCCTCCTG
GCCGGGGGTGGAAAGTTCTTCGGTTGGGGATGGGCTCGCGGCCTATCAGCTTGTTGGT
GGGGTAACGGCCTACCAAGGCGATTACGGGTAGCCGGCCTGAGAGGGCGACCGGCCA
CACTGGGACTGAGACACGGCCCAGACTCCTGCGGGAGGCAGCAGTGGGGAATATTGC
GCAATGGGCGAAAGCCTGACGCAGCGACGCCGCGTGGGGGATGACGGCCTTCGGGTT
GTAAACCTCTTTACCACCAACGCAGGCTCGGAGTTCTCTTCGGGTTGACGGTAGGTG
GGGAATAAGGACCGGCTAACTACGTGCCAGCAGCCGCGGTAATACGTAGGGTCCGAGC
GTTGTCCGGAATTATTGGGCGTAAAGAGCTCGTAGGCGGCGTGTCGCGTCTGCTGTGA
AAGACCGGGGCTTAACTCCGGTTTGGCAGTGGATACGGGCATGCTAGAGGTAGGTAGG
GGAGACTGGAATTCCTGGTGTAGCGGTGAAATGCGCAGATATCAGGAGGAACACCGGT
GGCGAAGGCGGGTCTCTGGGCCTTACCTGACGCTGAGGAGCGAAAGCATGGGGAGCG
AACAGGATTAGATACCCTGGTAGTCCATGCCGTAAACGTTGGGCGCTAGGTGTGGGGA
CTTTCCACGGTTTCCGCGCCGTAGCTAACGCATTAAGCGCCCCGCCTGGGGAGTACGG
CCGCAAGGCTAAAACTCAAAGGAATTGACGGGGGCCCGCACAAGCGGCGGAGCATGT
TGCTTAATTCGACGCAACGCGAAGAACCTTACCAAGGTTTGACATCACCCGTGGACTC
GCAGAGATGTGAGGTCATTTGGTTGGCGGGTGACAGGTGGTGCATGGCTGTCGTCAGC
TCGTGTCGTGAGATGTTGGGTTAAGTCCCGCAACGAGCGCAACCCTTGTTCCATGTTG
CCAGCACGTACTGGTGGGGACTCATGGGAGACTGCCGGGGTCAACTCGGAGGAAGGT
GGGGATGACGTCAAGTCATCATGCCCCTTATGTCTTGGGCTGCAAACATGCTACAATGG
CCGGTACAATGGGCGTGCGATGCCGTAAGGTGGAGCGAATCCCTAAAAGCCGGTCTCA

GTTCGGATTGGGGTCTGCAACTCGACCCCATGAAGGTGGAGTCGCTAGTAATCGCGGA
TCAGCAACGCCGCGGTGAATACGTTCCCGGGCCTTGTACACACCGCCCGTCACGTCAT
GAAAGTCGGCAACACCCGAAACTTGCGGCCTAACCCCTTGTGGGAGGGAGTGAGTGA
AGGTGGGGCTGGCGATTGGGACGAAGTCGTAACAAGGTAACCGTACCGGAAGGTGCG
GCTGGA

>KU860457.1 Nocardiopsis akesuensis strain TRM 46250 16S ribosomal RNA gene, partial sequence

CGAGCGGCGAACGGGTGAGTAACACGTGAGTAACCTGCCCCTGACTCTGGGATAAGC
CGTGGAAACGCGGTCTAATACCGGATATGACACCGTGCCGCATGGCGGGGTGTGGAAA
GTTGTTTCGGTTGGGGATGGACTCGCGGCCTATCAGCTTGTTGGTGGGGTAAAGGCCT
ACCAAGGCGATTACGGGTAGCCGGCCTGAGAGGGCGACCGGCCACACTGGGACTGAG
ACACGGCCCAGACTCCTGCGGGAGGCAGCAGTGGGGAATATTGCGCAATGGGCGGAA
GCCTGACGCAGCGACGCCGCGTGGGGGATGACGGCCTCCGGGTTGTAAACCTCTTTTA
CCACTCACGCAGGCCCGGGGTTTTCTTCGGGTTGACGGTAGGTGGGGAATAAGGACCG
GCTAACTACGTGCCAGCAGCCGCGGTAATACGTAGGGTCCGAGCGTTGTCCGGAATTAT
TGGGCGTAAAGAGCTCGTAGGCGGTGTGTCGCGTCTGCTGTGAAAGGCTGGGGCTCA
ACCCTGGTTTTGCAGTGGATACGGGCATGCTAGAGGTAGGTAGGGGAGACTGGAATTC
CTGGTGTAGCGGTGAAATGCGCAGATATCAGGAGGAACACCGGTGGCGAAGGCGGGT
CTCTGGGCCTTACCTGACGCTGAGGAGCGAAAGCGTGGGGAGCGAACAGGATTAGAT
ACCCTGGTAGTCCACGCCGTAAACGTTGGGCGCTAGGTGTGGGGGCTTTCCACGGTTC
CCGTGCCGCAGCTAACGCATTAAGCGCCCCGCCTGGGGAGTACGGCCGCAAGGCTAA
AACTCAGAGGAATTGACGGGGGCCCGCACAAGCGGCGGAGCATGTTGCTTAATTCGA
CGCAACGCGAAGAACCTTACCAAGGTTTGACATCACCGGTAATCCGTTAGAGATAGCG
GGTCCTTCGGGGATCGGTGACAGGTGGTGCATGGCTGTCGTCAGCTCGTGTCGTGAGA
TGTTGGGTTAAGTCCCGCAACGAGCGCAACCCTTGTTCCATGTTGCCAGCACGTGATG
GTGGGGACTCATGGGAGACTGCCGGGGTCAACTCGGAGGAAGGTGGGGATGACGTCA
AGTCATCATGCCCCTTATGTCTTGGGCTGCAAACATGCTACAATGGCCGGTACAGTGGG
CTTGCGATGCCGTGAGGTGGAGCGAATCCCTAAAAGCCGGTCTCAGTTCGGATTGGGG
TCTGCAACTCGACCCCATGAAGGTGGAGTCGCTAGTAATCGCGGATCAGCATTGCCGC
GGTGAATACGTTCCCGGGCCTTGTACACACCGCCCGTCACGTCATGAAAGTCGGCAAC
ACCCGAAACCTGTGGCCTAACCCCTTGTGGGAGGGAGTGGGTGAAGGTGGGGCTGGC
GATTGGGACGAAGTCGTAACAAGGTAGCC

>OK299036.1 Nocardiopsis aegyptia strain TRM86122 16S ribosomal RNA gene, partial sequence

GGGCGCGTGCTTACCTGCAGTCGAGCGGTAAGGCCCTTCGGGGTACACGAGCGGCGA
ACGGGTGAGTAACACGTGAGCAACCTGCCCCCGACTCCGGGATAAGCGGTGGAAACG
CCGTCTAATACCGGATACGACCGGCCACCTCATGGTAGCCGGTGGAAAGTTTTTCGGTT
GGGGATGGGCTCGCGGCCTATCAGCTTGTTGGTGGGGTAAAGGCCTACCAAGGCGATT
ACGGGTAGCCGGCCTGAGAGGGCGACCGGCCACACTGGGACTGAGACACGGCCCAG
ACTCCTGCGGGAGGCAGCAGTGGGGAATATTGCGCAATGGGCGAAAGCCTGACGCAG
CGACGCCGCGTGGGGGATGACGGCCTTCGGGTTGTAAACCTCTTTTACCACCAACGCA
GGCTCCCAGTTCTCTGGGGGTTGACGGTAGGTGGGGAATAAGGACCGGCTAACTACGT
GCCAGCAGCCGCGGTAATACGTAGGGTCCGAGCGTTGTCCGGAATTATTGGGCGTAAA
GAGCTCGTAGGCGGCGTGTCGCGTCTGCTGTGAAAGACCGGGGCTTAACTCCGGTTCT

GCAGTGGATACGGGCATGCTAGAGGTAGGTAGGGGAGACTGGAATTCCTGGTGTAGCG
GTGAAATGCGCAGATATCAGGAGGAACACCGGTGGCGAAGGCGGGTCTCTGGGCCTT
ACCTGACGCTGAGGAGCGAAAGCATGGGGAGCGAACAGGATTAGATACCCTGGTAGT
CCATGCCGTAAACGTTGGGCGCTAGGTGTGGGGACTTTCCACGGTTTCCGCGCCGTAG
CTAACGCATTAAGCGCCCCGCCTGGGGAGTACGGCCGCAAGGCTAAAACTCAAAGGA
ATTGACGGGGGCCCGCACAAGCGGCGGAGCATGTTGCTTAATTCGACGCAACGCGAA
GAACCTTACCAAGGTTTGACATCACCCGTGGACCTGCAGAGATGTGGGGTCATTTAGT
TGGTGGGTGACAGGTGGTGCATGGCTGTCGTCAGCTCGTGTCGTGAGATGTTGGGTTA
AGTCCCGCAACGAGCGCAACCCTTATTCCATGTTGCCAGCACGTAGTGGTGGGGACTC
ATGGGAGACTGCCGGGGTCAACTCGGAGGAAGGTGGGGACGACGTCAAGTCATCATG
CCCCTTATGTCTTGGGCTGCAAACATGCTACAATGGCCGGTACAATGGGCGTGCGATAC
CGTAAGGTGGAGCGAATCCCTAAAAGCCGGTCTCAGTTCGGATTGGGGTCTGCAACTC
GACCCCATGAAGGTGGAGTCGCTAGTAATCGCGGATCAGCAACGCCGCGGTGAATACG
TTCCCGGGCCTTGTACACACCGCCCGTCACGTCATGAAAGTCGGCAACACCCGAAACT
TGTGGCCTAACCCTTCGGGGAGGGAATGAGGAAGGTGACTTA
>NR_026340.1 Nocardiopsis alba DSM 43377 16S ribosomal RNA, partial sequence
CGCTGGCGGCGTGCTTAACACATGCAAGTCGAGCGGTAAGGCCCTTCGGGGTACACGA
GCGGCGAACGGGTGAGTAACACGTGAGCAACCTGCCCCTGACTCTGGGATAAGCGGT
GGAAACGCCGTCTAATACCGGATACGACCTTCCGCCTCATGGTGGAGGGTGGAAAGTT
TTTTCGGTCAGGGATGGGCTCGCGGCCTATCAGCTTGTTGGTGGGGTAACGGCCTACC
AAGGCGATTACGGGTAGCCGGCCTGAGAGGGCGACCGGCCACACTGGGACTGAGACA
CGGCCCAGACTCCTGCGGGAGGCAGCAGTGGGGAATATTGCGCAATGGGCGAAAGCC
TGACGCAGCGACGCCGCGTGGGGGATGACGGCCTTCGGGTTGTAAACCTCTTTACCA
CCAACGCAGGCTCCGGGTTCTCTCGGGGTTGACGGTAGGTGGGGAATAAGGACCGGCT
AACTACGTGCCAGCAGCCGCGGTAATACGTAGGGTCCGAGCGTTGTCCGGAATTATTG
GGCGTAAAGAGCTCGTAGGCGGCGTGTCGCGTCTGCTGTGAAAGACCGGGGCTTAAC
TCCGGTTCTGCAGTGGATACGGGCATGCTAGAGGTAGGTAGGGGAGACTGGAATTCCT
GGTGTAGCGGTGAAATGCGCAGATATCAGGAGGAACACCGGTGGCGAAGGCGGGTCT
CTGGGCCTTACCTGACGCTGAGGAGCGAAAGCATGGGGAGCGAACAGGATTAGATAC
CCTGGTAGTCCATGCCGTAAACGTTGGGCGCTAGGTGTGGGGACTTTCCACGGTTTCC
GCGCCGTAGCTAACGCATTAAGCGCCCCGCCTGGGGAGTACGGCCGCAAGGCTAAAAC
TCAAAGGAATTGACGGGGGCCCGCACAAGCGGCGGAGCATGTTGCTTAATTCGACGC
AACGCGAAGAACCTTACCAAGGTTTGACATCACCCGTGGACCTGTAGAGATACAGGGT
CATTTAGTTGGTGGGTGACAGGTGGTGCATGGCTGTCGTCAGCTCGTGTCGTGAGATG
TTGGGTTAAGTCCCGCAACGAGCGCAACCCTTGTTCCATGTTGCCAGCACGTAATGGT
GGGGACTCATGGGAGACTGCCGGGGTCAACTCGGAGGAAGGTGGGGACGACGTCAA
GTCATCATGCCCCTTATGTCTTGGGCTGCAAACATGCTACAATGGCCGGTACAATGGGC
GTGCGATACCGTAAGGTGGAGCGAATCCCTTAAAGCCGGTCTCAGTTCGGATTGGGGT
CTGCAACTCGACCCCATGAAGGTGGAGTCGCTAGTAATCGCGGATCAGCAACGCCGCG
GTGAATACGTTCCCGGGCCTTGTACACACCGCCCGTCACGTCATGAAAGTCGGCAACA
CCCGAAACTTGCGGCCTAACCCTTCGGGGAGGGAGTGAGTGAAGGTGGGGCTGGCGA
TTGGGACGAAGTCGTAACAA
>NR_029343.1 Nocardiopsis synnemataformans strain IMMIB D-1215 16S ribosomal RNA, partial sequence

CGCTGGCGGCGTGCTTAACACATGCAAGTCGAGCGGTAAGGCCCTTCGGGGTACACGA
GCGGCGAACGGGTGAGTAACACGTGAGCAACCTGCCCCTGACTCTGGGATAAGCGGT
GGAAACGCCGTCTAATACCGGATACGACCTGCCACCTCATGGTGGAGGGTGGAAAGTT
TTTCGGTCAGGGATGGGCTCGCGGCCTATCAGCTTGTTGGTGGGGTAACGGCCTACCA
AGGCGATTACGGGTAGCCGGCCTGAGAGGGCGACCGGCCACACTGGGACTGAGACAC
GGCCCAGACTCCTGCGGGAGGCAGCAGTGGGGAATATTGCGCAATGGGCGAAAGCCT
GACGCAGCGACGCCGCGTGGGGGATGACGGCCTTCGGGTTGTAAACCTCTTTTACCAC
CAACGCAGGCTCGAAGTTTTCTTCGGGTTGACGGTAGGTGGGGAATAAGGACCGGCTA
ACTACGTGCCAGCAGCCGCGGTAATACGTAGGGTCCGAGCGTTGTCCGGAATTATTGG
GCGTAAAGAGCTCGTAGGCGGCGTGTCGCGTCTGCTGTGAAAGACCGGGGCTTAACTC
CGGTTCTGCAGTGGATACGGGCATGCTAGAGGTAGGTAGGGGAGACTGGAATTCCTGG
TGTAGCGGTGAAATGCGCAGATATCAGGAGGAACACCGGTGGCGAAGGCGGGTCTCT
GGGCCTTACCTGACGCTGAGGAGCGAAAGCATGGGGAGCGAACAGGATTAGATACCC
TGGTAGTCCATGCCGTAAACGTTGGGCGCTAGGTGTGGGGACTTTCCACGGTTTCCGC
GCCGTAGCTAACGCATTAAGCGCCCCGCCTGGGGAGTACGGCCGCAAGGCTAAAACTC
AAAGGAATTGACGGGGGCCCGCACAAGCGGCGGAGCATGTTGCTTAATTCGACGCAA
CGCGAAGAACCTTACCAAGGTTTGACATCACCCGTGGACTCGCAGAGATGTGAGGTCA
TTTAGTTGGCGGGTGACAGGTGGTGCATGGCTGTCGTCAGCTCGTGTCGTGAGATGTT
GGGTTAAGTCCCGCAACGAGCGCAACCCTTGTTCCATGTTGCCAGCACGTAATGGTGG
GGACTCATGGGAGACTGCCGGGGTCAACTCGGAGGAAGGTGGGGATGACGTCAAGTC
ATCATGCCCCTTATGTCTTGGGCTGCAAACATGCTACAATGGCCGGTACAATGGGCGTG
CGATACCGCAAGGTGGAGCGAATCCCTAAAAGCCGGTCTCAGTTCGGATTGGGGTCTG
CAACTCGACCCCATGAAGGTGGAGTCGCTAGTAATCGCGGATCAGCAACGCCGCGGTG
AATACGTTCCCGGGCCTTGTACACACCGCCCGTCACGTCATGAAAGTCGGCAACACCC
GAAACTTGCGGCCTAACCCCTTGTGGGAGGGAGTGAGTGAAGGTGGGGCTGGCGATT
GGGACGACGTCGTAACAA
>NR_024958.1 Nocardiopsis trehalosi strain VKM Ac-942 16S ribosomal RNA, partial sequence
ATCCTGGCTCAGGACGAACGCTGGCGGCGTGCTTAACACATGCAAGTCGAGCGGTAA
GGCCCTTCGGGGTACACGAGCGGCGAACGGGTGAGTAACACGTGAGTAACCTGCCCT
TGACTCTGGGATAAGCCGGGGAAACCCGGTCTAATACCGGATAGGACACCCTTCCGCA
TGGTGGGGTGTGGAAAGTTTTTCGGTCAGGGATGGACTCGCGGCCTATCAGCTTGTTG
GTGGGGTAACGGCCTACCAAGGCGATTACGGGTAGCCGGCCTGAGAGGGCGACCGGC
CACACTGGGACTGAGACACGGCCCAGACTCCTGCGGGAGGCAGCAGTGGGGAATATT
GCGCAATGGGCGAAAGCCTGACGCAGCGACGCCGCGTGGGGGATGACGGCCTTCGGG
TTGTAAACCTCTTTTACCACTCACGCAGGCCGGGGGTTTTCTCCCGGTTGACGGTAGGT
GGGGAATAAGGACCGGCTAACTACGTGCCAGCAGCCGCGGTAATACGTAGGGTCCGAG
CGTTGTCCGGAATTATTGGGCGTAAAGAGCTCGTAGGCGGCGTGTCGCGTCTGCTGTG
AAAGACCGGGGCTTAACTCCGGTTTTGCAGTGGATACGGGCATGCTAGAGGTAGGTAG
GGGAGACTGGAATTCCTGGTGTAGCGGTGAAATGCGCAGATATCAGGAGGAACACCG
GTGGCGAAGGCGGGTCTCTGGGCCTTACCTGACGCTGAGGAGCGAAAGCGTGGGGAG
CGAACAGGATTAGATACCCTGGTAGTCCACGCCGTAAACGTTGGGCGCTAGGTGTGGG
GACTTTCCACGGTCTCCGTGCCGCAGCTAACGCATTAAGCGCCCCGCCTGGGGAGTAC
GGCCGCAAGGCTAAAACTCAAAGGAATTGACGGGGGCCCGCACAAGCGGCGGAGCAT
GTTGCTTAATTCGACGCAACGCGAAGAACCTTACCAAGGTTTGACATCACCGGTAATC

CCGCAGAGATGCGGGGTCCTTTTGGGATCGGTGACATGTGGTGCATGGCTGTCGTCAG
CTCGTGTCGTGAGATGTTGGGTTAAGTCCCGCAACGAGCGCAACCCTTGTTCCATGTT
GCCAGCACGTAGTGGTGGGGACTCATGGGAGACTGCCGGGGTCAACTCGGAGGAAGG
TGGGGATGACGTCAAGTCATCATGCCCCTTATGTCTTGGGCTGCAAACATGCTACAATG
GCCGGTACAATGGGCGTGCGATACCGTGAGGTGGAGCGAATCCCTAAAAGCCGGTCTC
AGTTCGGATTGGGGTCTGCAACTCGACCCCATGAAGGTGGAGTCGCTAGTAATCGCGG
ATCAGCAACGCCGCGGTGAATACGTTCCCGGGCCTTGTACACACCGCCCGTCACGTCA
TGAAAGTCGGCAACACCCGAAACTTGTGGCCTAACCCTTCGGGGAGGGAGCGAGTGA
AGGTGGGGCTGGCGATTGGGACGAAGTCGTAA
>AY230848.1 Nocardiopsis egyptensis 16S ribosomal RNA gene, partial sequence
CRATDBYDNACTBDATMBDNASTNCTTTTATCTTGGCTCAGGACGAACGCTGGCGGCG
TGCTTAACACATGCAAGTCGAGCGGTAAGCCCTTCGGGGTACACGAGCGGCGAACGG
GTGAGTAACACGTGAGCAACCTGCCCCCGACTCCGGGATAAGCGGTGGAAACGCCGT
CTAATACCGGATACGACCCGTCACCTCATGGTGTGCGGGTGGAAAGTTTTTCGGTTGG
GGATGGGCTCGCGGCCTATCAGCTTGTTGGTGGGGTAACGGCCCACCAAGGCGATTAC
GGGTAGCCGGCCTGAGAGGGCGACCGGCCACACTGGGACTGAGACACGGCCCAGACT
CCTACGGGAGGCAGCAGTGGGGAATATTGCACAATGGGCGCAAGCCTGATGCAGCGA
CGCCGCGTGGGGGATGACGGCCTTCGGGTTGTAAACCTCTTTTACCACTCACGCAGGC
TCCACGTTCTCGTGGGGTTGACGGTCAGTGGGGAATAAGGACCGGCTAACTACGTGCC
AGCAGCCGCGGTAATACGTAGGGTCCGAGCGTTGTCCGGAATTATTGGGCGTAAAGAG
CTCGTAGGCGGCATGTCGCGTCTGCTGTGAAAGACCGGGGCTTAACTCCGGTTCTGCA
GTGGATACGGGCATGCTAGAGGTAGGTAGGGGAGACTGGAATTCCTGGTGTAGCGGTG
AAATGCGCAGATATCAGGAGGAACACCGGTGGCGAAGGCGGGTCTCTGGGCCTTACCT
GACGCTGAGGAGCGAAAGCATGGGTAGCGAACAGGATTAGATACCCTGGTAGTCCATG
CCGTAAACGTTGGGCGCTAGGTGTGGGGACTTTCCACGGTTTCCGCGCCGTAGCTAAC
GCATTAAGCGCCCCGCCTGGGGAGTACGGCCGCAAGGCTAAAACTCAAAGGAATTGA
CGGGGGCCCGCACAAGCGGCGGAGCATGTTGCTTAATTCGACGCAACGCGAAGAAC
CTTACCAAGGTTTGACATCACCCGTGGACCTGTAGAGATACAGGGTCATTTGGTTGGCG
GGTGACAGGTGGTGCATGGCTGTCGTCAGCTCGTGTCGTGAGATGTTGGGTTAAGTCC
CGCAACGAGCGCAACCCTTGTTCCATGTTGCCAGCACGTAGTGGTGGGGACTCATGGG
AGACTGCCGGGGTCAACTCGGAGGAAGGTGGGGACGACGTCAAGTCATCATGCCCCT
TATGTCTTGGGCTGCAAACATGCTACAATGGCCGGTACAATGGGCGTGCGATACCGTGA
GGTGGAGCGAATCCCTGAAAGCCGGTCTCAGTTCGGATTGGGGTCTGCAACTCGACCC
CATGAAGGTGGAGTCGCTAGTAATCGCGGATCAGCAACGCCGCGGTGAATACGTTCCC
GGGCCTTGTACACACCGCCCGTCACGTCATGAAAGTCGGCAACACCCGAAACTTGTGG
CCTAACCCTTCGGGGAGGGAATGAGTGAAGGTGGGGCTGGCGATTGGGACGAAGTCG
TAACAAGGTAGCCG
>MN004803.1 Nocardiopsis lucentensis strain LUPA60-89 16S ribosomal RNA gene, partial sequence
CGCTGGCGGCGTGCTTAACACATGCAAGTCGAGCGGTAAGGCCCTTCGGGGTACACGA
GCGGCGAACGGGTGAGTAACACGTGAGCAACCTGCCCCTGACTCCGGGATAAGCGGT
GGAAACGCCGTCTAATACCGGATACGACCCACCACCTCATGGTGGAGGGTGGAAAGTT
TTTCGGTCAGGGATGGGCTCGCGGCCTATCAGCTTGTTGGTGGGGTAACGGCCTACCA
AGGCGATTACGGGTAGCCGGCCTGAGAGGGCGACCGGCCACACTGGGACTGAGACAC

GGCCCAGACTCCTGCGGGAGGCAGCAGTGGGGAATATTGCGCAATGGGCGAAAGCCT
GACGCAGCGACGCCGCGTGGGGGATGACGGCCTTCGGGTTGTAAACCTCTTTACCAC
CAACGCAGGCTCCGGGTTCTCCCGGGGTTGACGGTAGGTGGTGAATAAGGACCGGCTA
ACTACGTGCCAGCAGCCGCGGTAATACGTAGGGTCCGAGCGTTGTCCGGAATTATTGG
GCGTAAAGAGCTCGTAGGCGGCGTGTCGCGTCTGCTGTGAAAGACCGGGGCTTAACTC
CGGTTCTGCAGTGGATACGGGCATGCTAGAGGTAGGTAGGGGAGACTGGAATTCCTGG
TGTAGCGGTGAAATGCGCAGATATCAGGAGGAACACCGGTGGCGAAGGCGGGTCTCT
GGGCCTTACCTGACGCTGAGGAGCGAAAGCATGGGGAGCGAACAGGATTAGATACCC
TGGTAGTCCATGCCGTAAACGTTGGGCGCTAGGTGTGGGGACTTTCCACGGTTTCCGC
GCCGTAGCTAACGCATTAAGCGCCCCGCCTGGGGAGTACGGCCGCAAGGCTAAAACTC
AAAGGAATTGACGGGGGCCCGCACAAGCGGCGGAGCATGTTGCTTAATTCGACGCAA
CGCGAAGAACCTTACCAAGGTTTGACATCACCCGTGGACCTGCAGAGATGTGGGGTCA
TTTAGTTGGTGGGTGACAGGTGGTGCATGGCTGTCGTCAGCTCGTGTCGTGAGATGTT
GGGTTAAGTCCCGCAACGAGCGCAACCCTTGTTCCATGTTGCCAGCACGTAATGGTGG
GGACTCATGGGAGACTGCCGGGGTCAACTCGGAGGAAGGTGGGGACGACGTCAAGTC
ATCATGCCCCTTATGTCTTGGGCTGCAAACATGCTACAATGGCCGGTACAATGGGCGTG
CGATACCGTGAGGTGGAGCGAATCCCTAAAAGCCGGTCTCAGTTCGGATTGGGGTCTG
CAACTCGACCCCATGAAGGTGGAGTCGCTAGTAATCGCGGATCAGCAACGCCGCGGTG
AATACGTTCCCGGGCCTTGTACACACCGCCCGTCACGTCATGAAAGTCGGCAACACCC
GAAACTTGTGGCCCAACCCCTTGTGGGAGGGAATGAGTGAAGGTGGGGCTGGCGATT
GGGACGAAGTCGTAACAA
>KF146896.1 Nocardiopsis dassonvillei strain BA6-3 16S ribosomal RNA gene, partial sequence
AACGTGGGGGCGTGCTTACACATGCAAGTCGAGCGGTAAGGCCCTTCGGGGTACACG
AGCGGCGAACGGGTGAGTAACACGTGAGCAACCTGCCCCTGACTCCGGGATAAGCGG
TGGAAACGCCGTCTAATACCGGATACGACCCGCCACCTCATGGTGGAGGGTGGAAAGT
TTTTCGGTCAGGGATGGGCTCGCGGCCTATCAGCTTGTTGGTGGGGTAACGGCCTACC
AAGGCGATTACGGGTAGCCGGCCTGAGAGGGCGACCGGCCACACTGGGACTGAGACA
CGGCCCAGACTCCTGCGGGAGGCAGCAGTGGGGAATATTGCGCAATGGGCGAAAGCC
TGACGCAGCGACGCCGCGTGGGGGATGACGGCCTTCGGGTTGTAAACCTCTTTACCA
CCAACGCAGGCTCCCAGTTCTCTGGGGGTTGACGGTAGGTGGGGAATAAGGACCGGCT
AACTACGTGCCAGCAGCCGCGGTAATACGTAGGGTCCGAGCGTTGTCCGGAATTATTG
GGCGTAAAGAGCTCGTAGGCGGCGTGTCGCGTCTGCTGTGAAAGACCGGGGCTTAAC
TCCGGTTCTGCAGTGGATACGGGCATGCTAGAGGTAGGTAGGGGAGACTGGAATTCCT
GGTGTAGCGGTGAAATGCGCAGATATCAGGAGGAACACCGGTGGCGAAGGCGGGTCT
CTGGGCCTTACCTGACGCTGAGGAGCGAAAGCATGGGGAGCGAACAGGATTAGATAC
CCTGGTAGTCCATGCCGTAAACGTTGGGCGCTAGGTGTGGGGACTTTCCACGGTTTCC
GCGCCGTAGCTAACGCATTAAGCGCCCCGCCTGGGGAGTACGGCCGCAAGGCTAAAAC
TCAAAGGAATTGACGGGGGCCCGCACAAGCGGCGGAGCATGTTGCTTAATTCGACGC
AACGCGAAGAACCTTACCAAGGTTTGACATCACCCGTGGACTCGCAGAGATGTGAGG
TCATTTAGTTGGCGGGTGACAGGTGGTGCATGGCTGTCGTCAGCTCGTGTCGTGAGAT
GTTGGGTTAAGTCCCGCAACGAGCGCAACCCTTGTTCCATGTTGCCAGCACGTAATGG
TGGGGACTCATGGGAGACTGCCGGGGTCAACTCGGAGGAAGGTGGGGATGACGTCAA
GTCATCATGCCCCTTATGTCTTGGGCTGCAAACATGCTACAATGGCCGGTACAATGGGC
GTGCGATACCGTAAGGTGGAGCGAATCCCTAAAAGCCGGTCTCAGTTCGGATTGGGGT

CTGCAACTCGACCCCATGAAGGTGGAGTCGCTAGTAATCGCGGATCAGCAACGCCGCG
GTGAATACGTTCCCGGGCCTTGTACACACCGCCCGTCACGTCATGAAAGTCGGCAACA
CCCGAAACTTGCGGCCTAACCCCTTGTGGGAGGGAGTGAGTGAAGGTGGCCCGGGGT
C
>MN049987.1 Nocardiopsis flavescens strain 205524 16S ribosomal RNA gene, partial sequence
GCAATGGCGGCGTGCTTACACATGCAAGTCGAGCGGTAAGGCCCTTCGGGGTACACGA
GCGGCGAACGGGTGAGTAACACGTGAGCAACCTGCCCCTGACTCCGGGATAAGCGGT
GGAAACGCCGTCTAATACCGGATACGACCCTCCACCTCATGGTGGTGGGTGGAAAGTT
TTTCGGTCGGGGATGGGCTCGCGGCCTATCAGCTTGTTGGTGGGGTAACGGCCTACCA
AGGCGATTACGGGTAGCCGGCCTGAGAGGGCGACCGGCCACACTGGGACTGAGACAC
GGCCCAGACTCCTGCGGGAGGCAGCAGTGGGGAATATTGCGCAATGGGGAAAGCCTG
ACGCAGCGACGCCGCGTGGGGGATGACGGCCTTCGGGTTGTAAACCTCTTTTACCACT
CACGCAGGCTCCACGTTCTCGTGGGGTTGACGGTAGGTGGGGAATAAGGACCGGCTA
ACTACGTGCCAGCAGCCGCGGTAATACGTAGGGTCCGAGCGTTGTCCGGAATTATTGG
GCGTAAAGAGCTCGTAGGCGGCGTGTCGCGTCTGCTGTGAAAGACCGGGGCTTAACC
CCGGTTCTGCAGTGGATACGGGCATGCTAGAGGTAGGTAGGGGAGACTGGAATTCCTG
GTGTAGCGGTGAAATGCGCAGATATCAGGAGGAACACCGGTGGCGAAGGCGGGTCTC
TGGGCCTTACCTGACGCTGAGGAGCGAAAGCATGGGGAGCGAACAGGATTAGATACC
CTGGTAGTCCATGCCGTAAACGTTGGGCGCTAGGTGTGGGGACTTTCCACGGTTTCCG
CGCCGTAGCTAACGCATTAAGCGCCCCGCCTGGGGAGTACGGCCGCAAGGCTAAAACT
CAAAGGAATTGACGGGGGCCCGCACAAGCGGCGGAGCATGTTGCTTAATTCGACGCA
ACGCGAAGAACCTTACCAAGGTTTGACATCACCCGTGGACCTGCAGAGATGTGGGGTC
ATTTAGTTGGCGGGTGACAGGTGGTGCATGGCTGTCGTCAGCTCGTGTCGTGAGATGT
TGGGTTAAGTCCCGCAACGAGCGCAACCCTTGTTCCATGTTGCCAGCACGTAATGGTG
GGGACTCATGGGAGACTGCCGGGGTCAACTCGGAGGAAGGTGGGGACGACGTCAAG
TCATCATGCCCCTTATGTCTTGGGCTGCAAACATGCTACAATGGCCGGTACAATGGGCG
TGCGATACCGTGAGGTGGAGCGAATCCCTAAAAGCCGGTCTCAGTTCGGATTGGGTCT
GCAACTCGACCCCATGAAGGTGGAGTCGCTAGTAATCGCGGATCAGCAACGCCGCGGT
GAATACGTTCCCGGGCCTTGTACACACCGCCCGTCACGTCATGAAAGTCGGCAACACC
CGAAACTTGTGGCCTAACCCCTTGTGGGAGGGAATGAGGAAGGTGGCGCAATT
>MN004803.1 Nocardiopsis lucentensis strain LUPA60-89 16S ribosomal RNA gene, partial sequence
CGCTGGCGGCGTGCTTAACACATGCAAGTCGAGCGGTAAGGCCCTTCGGGGTACACGA
GCGGCGAACGGGTGAGTAACACGTGAGCAACCTGCCCCTGACTCCGGGATAAGCGGT
GGAAACGCCGTCTAATACCGGATACGACCCACCACCTCATGGTGGAGGGTGGAAAGTT
TTTCGGTCAGGGATGGGCTCGCGGCCTATCAGCTTGTTGGTGGGGTAACGGCCTACCA
AGGCGATTACGGGTAGCCGGCCTGAGAGGGCGACCGGCCACACTGGGACTGAGACAC
GGCCCAGACTCCTGCGGGAGGCAGCAGTGGGGAATATTGCGCAATGGGCGAAAGCCT
GACGCAGCGACGCCGCGTGGGGGATGACGGCCTTCGGGTTGTAAACCTCTTTTACCAC
CAACGCAGGCTCCGGGTTCTCCCGGGGTTGACGGTAGGTGGTGAATAAGGACCGGCTA
ACTACGTGCCAGCAGCCGCGGTAATACGTAGGGTCCGAGCGTTGTCCGGAATTATTGG
GCGTAAAGAGCTCGTAGGCGGCGTGTCGCGTCTGCTGTGAAAGACCGGGGCTTAACTC
CGGTTCTGCAGTGGATACGGGCATGCTAGAGGTAGGTAGGGGAGACTGGAATTCCTGG
TGTAGCGGTGAAATGCGCAGATATCAGGAGGAACACCGGTGGCGAAGGCGGGTCTCT

GGGCCTTACCTGACGCTGAGGAGCGAAAGCATGGGGAGCGAACAGGATTAGATACCC
TGGTAGTCCATGCCGTAAACGTTGGGCGCTAGGTGTGGGGACTTTCCACGGTTTCCGC
GCCGTAGCTAACGCATTAAGCGCCCCGCCTGGGGAGTACGGCCGCAAGGCTAAAACTC
AAAGGAATTGACGGGGGCCCGCACAAGCGGCGGAGCATGTTGCTTAATTCGACGCAA
CGCGAAGAACCTTACCAAGGTTTGACATCACCCGTGGACCTGCAGAGATGTGGGGTCA
TTTAGTTGGTGGGTGACAGGTGGTGCATGGCTGTCGTCAGCTCGTGTCGTGAGATGTT
GGGTTAAGTCCCGCAACGAGCGCAACCCTTGTTCCATGTTGCCAGCACGTAATGGTGG
GGACTCATGGGAGACTGCCGGGGTCAACTCGGAGGAAGGTGGGGACGACGTCAAGTC
ATCATGCCCCTTATGTCTTGGGCTGCAAACATGCTACAATGGCCGGTACAATGGGCGTG
CGATACCGTGAGGTGGAGCGAATCCCTAAAAGCCGGTCTCAGTTCGGATTGGGGTCTG
CAACTCGACCCCATGAAGGTGGAGTCGCTAGTAATCGCGGATCAGCAACGCCGCGGTG
AATACGTTCCCGGGCCTTGTACACACCGCCCGTCACGTCATGAAAGTCGGCAACACCC
GAAACTTGTGGCCCAACCCCTTGTGGGAGGGAATGAGTGAAGGTGGGGCTGGCGATT
GGGACGAAGTCGTAACAA

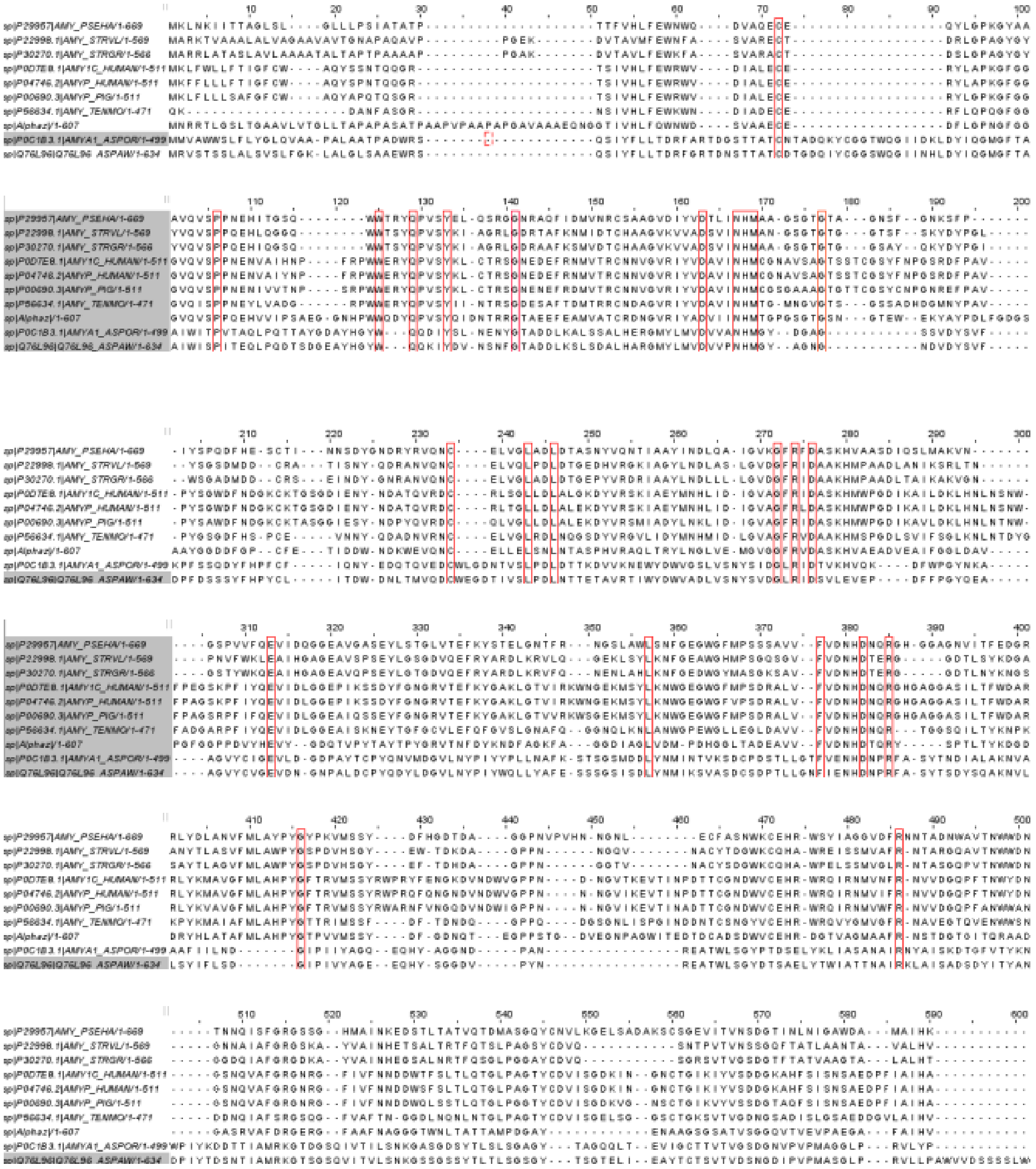

Figure S3. Sequence comparison of Alphaz with amylases from mammalian sources and Antarctic cold-adaptedsources

The base sequence of Alphaz:

ATGAACAGACGCACACTCGGCTCCCTCACCGGGGCCGCGGTGCTGGTCACCGGTCTCC
TCACGGCTCCGGCACCGGCGTCGGCCACCCCCGCGGCGCCCGTCCCCGCCGCACCCG
CCCCCGGAGCGGTCGCGGCCGCCGAGCAGAACGGCGGAACGATCGTCCACCTCTTCC
AGTGGAACTGGGACTCCGTGGCCGCCGAGTGCGAGGACTTCCTCGGCCCGAACGGGT
TCGGCGGCGTCCAGGTCTCCCCGCCCCAGGAGCACGTGGTCATCCCCTCCGCCGAGGG
CGGCAACCACCCCTGGTGGCAGGACTACCAGCCGGTCTCCTACCAGATCGACAACACC
CGGCGCGGCACGGCCGAGGAGTTCGAGGCCATGGTCGCCACGTGCCGCGACAACGGC
GTCCGGATCTACGCCGACGTCATCATCAACCACATGACCGGCCCCGGATCGGGCACCG
GAAGCAACGGCACCGAGTGGGAGAAGTACGCCTACCCGGACCTGTTCGGCGACGGCA

GCGCCGCCTACGGCGGCGACGACTTCGGCCCCTGCTTCGAGACGATCGACGACTGGA
ACGACAAGTGGGAGGTCCAGAACTGTGAACTCCTGGAGCTGTCGAACCTGAACACGG
CCTCCCCGCACGTGCGCGCACAGCTCACCCGCTACCTCAACGGGCTCGTGGAGATGGG
TGTGGGCGGCTTCCGCGTGGACGCCTCCAAGCACGTCGCCGAGGCCGACGTCGAGGC
GATCTTCGGCGGGCTCGACGCGGTCCCCGGATTCGGCGGACCGCCCGACGTCTACCAC
GAGGTCTACGGCGACCAGACGGTGCCCTACACCGCCTACACCCCGTACGGCCGGGTCA
CCAACTTCGACTACAAGAACGACTTCGCCGGGAAGTTCGCCGGCGGCGACATCGCCG
GACTGGTCGACATGCCCGACCACGGAGGCCTGACCGCCGACGAGGCCGTGGTGTTCG
TCGACAACCACGACACCCAGCGCTACAGCCCGACCCTCACCTACAAGGACGGCGACC
GCTACCACCTCGCCACCGCCTTCATGCTCGCCCACCCCTACGGCACCCCCGTGGTGATG
TCGAGCTACGACTTCGGCGACAACCAGACCGAGGGCCCGCCCAGCACCGGCGACGTC
GAGGGCAACCCGGCCGGATGGATCACCGAGGACACTGACTGCGCCGACTCCGACTGG
GTCTGCGAGCACCGCGACGGCACCGTCGCGGGGATGGCGGCCTTCCGCAACTCCACC
GACGGCACTGGGATCACCCAGCGCGCCGCGGACGGCGCCTCCCGCGTCGCCTTCGAC
CGCGGTGAGCGCGGCTTCGCGGCCTTCAACGCCGGCGGCGGCACCTGGAACCTGACC
GCCACCACGGCCATGCCCGACGGCGCGTACGAGAACGCGGCGGGCAGCGGCTCGGCC
ACCGTCTCCGGCGGACAGGTCACCGTGGAGGTCCCGGCCGAGGGTGCCTTCGCCATC
CACGTCGACGGCGTCTGCACCGACCCCGCCGAGTGCGACGGAGACGGAGACGGCGA
CGACGGAAACACCGTCCAGGTGTCCGCCCACGTCGAGACCTACTTCGGCCAGGAGGT
GTACATCGTCGGCGAGACCTCCGCGCTCGGCTCCTGGAACCCCGCGTCCGGCGCGCGG
CTCTCGACCGACGAGAGCACCTATCCCCAGTGGACCGGCGAGGCCGTCATCGGACCCG
ACGACGAGTGGAAGCTCGTCAAGATCGACGGCTCCGGCGCCGTCGAGTGGGAGTCCG
GCGCCAACCGCGTCGGCCCCGACACCGCCCCGGTCTGGCGGAACTGA

The amino acid sequences of α-amylase from other sources and the amino acid sequence of Alphaz used for sequence alignment:

>sp|P29957|AMY_PSEHA
MKLNKIITTAGLSLGLLLPSIATATPTTFVHLFEWNWQDVAQECEQYLGPKGYAAVQVSP
PNEHITGSQWWTRYQPVSYELQSRGGNRAQFIDMVNRCSAAGVDIYVDTLINHMAAGSG
TGTAGNSFGNKSFPIYSPQDFHESCTINNSDYGNDRYRVQNCELVGLADLDTASNYVQNTI
AAYINDLQAIGVKGFRFDASKHVAASDIQSLMAKVNGSPVVFQEVIDQGGEAVGASEYLS
TGLVTEFKYSTELGNTFRNGSLAWLSNFGEGWGFMPSSSAVVFVDNHDNQRGHGGAGN
VITFEDGRLYDLANVFMLAYPYGYPKVMSSYDFHGDTDAGGPNVPVHNNGNLECFASN
WKCEHRWSYIAGGVDFRNNTADNWAVTNWWDNTNNQISFGRGSSGHMAINKEDSTLTA
TVQTDMASGQYCNVLKGELSADAKSCSGEVITVNSDGTINLNIGAWDAMAIHKNAKLNT
SSASSTESDWQRTVIFINAQTQSGQDMFIRGGIDHAYANANLGRNCQTSNFECAMPIRHNN
LKNVTTSPWKANDNYLDWYGIENGQSSEAEGSATDWTTNVWPAGWGAEKTVNTDGFG
VTPLNIWGEHYWMLDVDMDCSKAVNGWFELKAFIKNGQGWETAIAQDNAPYTSTNHM
AQCGKINKFEFNNSGVVIRSF
>sp|P0C1B3.1|AMYA1_ASPOR
MMVAWWSLFLYGLQVAAPALAATPADWRSQSIYFLLTDRFARTDGSTTATCNTADQKYCG
GTWQGIIDKLDYIQGMGFTAIWITPVTAQLPQTTAYGDAYHGYWQQDIYSLNENYGTADD
LKALSSALHERGMYLMVDVVANHMGYDGAGSSVDYSVFKPFSSQDYFHPFCFIQNYEDQ
TQVEDCWLGDNTVSLPDLDTTKDVVKNEWYDWVGSLVSNYSIDGLRIDTVKHVQKDFW

PGYNKAAGVYCIGEVLDGDPAYTCPYQNVMDGVLNYPIYYPLLNAFKSTSGSMDDLYNM
INTVKSDCPDSTLLGTFVENHDNPRFASYTNDIALAKNVAAFIILNDGIPIIYAGQEQHYAG
GNDPANREATWLSGYPTDSELYKLIASANAIRNYAISKDTGFVTYKNWPIYKDDTTIAMR
KGTDGSQIVTILSNKGASGDSYTLSLSGAGYTAGQQLTEVIGCTTVTVGSDGNVPVPMAG
GLPRVLYPTEKLAGSKICSSS
>sp|P0DTE8.1|AMY1C_HUMAN
MKLFWLLFTIGFCWAQYSSNTQQGRTSIVHLFEWRWVDIALECERYLAPKGFGGVQVSPP
NENVAIHNPFRPWWERYQPVSYKLCTRSGNEDEFRNMVTRCNNVGVRIYVDAVINHMCG
NAVSAGTSSTCGSYFNPGSRDFPAVPYSGWDFNDGKCKTGSGDIENYNDATQVRDCRLSG
LLDLALGKDYVRSKIAEYMNHLIDIGVAGFRIDASKHMWPGDIKAILDKLHNLNSNWFPE
GSKPFIYQEVIDLGGEPIKSSDYFGNGRVTEFKYGAKLGTVIRKWNGEKMSYLKNWGEG
WGFMPSDRALVFVDNHDNQRGHGAGGASILTFWDARLYKMAVGFMLAHPYGFTRVMSS
YRWPRYFENGKDVNDWVGPPNDNGVTKEVTINPDTTCGNDWVCEHRWRQIRNMVNFR
NVVDGQPFTNWYDNGSNQVAFGRGNRGFIVFNNDDWTFSLTLQTGLPAGTYCDVISGDK
INGNCTGIKIYVSDDGKAHFSISNSAEDPFIAIHAESKL
>sp|P00690.3|AMYP_PIG
MKLFLLLSAFGFCWAQYAPQTQSGRTSIVHLFEWRWVDIALECERYLGPKGFGGVQVSPP
NENIVVTNPSRPWWERYQPVSYKLCTRSGNENEFRDMVTRCNNVGVRIYVDAVINHMCG
SGAAAGTGTTCGSYCNPGNREFPAVPYSAWDFNDGKCKTASGGIESYNDPYQVRDCQLV
GLLDLALEKDYVRSMIADYLNKLIDIGVAGFRIDASKHMWPGDIKAVLDKLHNLNTNWFP
AGSRPFIFQEVIDLGGEAIQSSEYFGNGRVTEFKYGAKLGTVVRKWSGEKMSYLKNWGE
GWGFMPSDRALVFVDNHDNQRGHGAGGASILTFWDARLYKVAVGFMLAHPYGFTRVMS
SYRWARNFVNGQDVNDWIGPPNNNGVIKEVTINADTTCGNDWVCEHRWRQIRNMVWF
RNVVDGQPFANWWANGSNQVAFGRGNRGFIVFNNDDWQLSSTLQTGLPGGTYCDVISG
DKVGNSCTGIKVYVSSDGTAQFSISNSAEDPFIAIHAESKL
>sp|P04746.2|AMYP_HUMAN
MKFFLLLFTIGFCWAQYSPNTQQGRTSIVHLFEWRWVDIALECERYLAPKGFGGVQVSPP
NENVAIYNPFRPWWERYQPVSYKLCTRSGNEDEFRNMVTRCNNVGVRIYVDAVINHMCG
NAVSAGTSSTCGSYFNPGSRDFPAVPYSGWDFNDGKCKTGSGDIENYNDATQVRDCRLTG
LLDLALEKDYVRSKIAEYMNHLIDIGVAGFRLDASKHMWPGDIKAILDKLHNLNSNWFPA
GSKPFIYQEVIDLGGEPIKSSDYFGNGRVTEFKYGAKLGTVIRKWNGEKMSYLKNWGEG
WGFVPSDRALVFVDNHDNQRGHGAGGASILTFWDARLYKMAVGFMLAHPYGFTRVMSS
YRWPRQFQNGNDVNDWVGPPNNNGVIKEVTINPDTTCGNDWVCEHRWRQIRNMVIFRN
VVDGQPFTNWYDNGSNQVAFGRGNRGFIVFNNDDWSFSLTLQTGLPAGTYCDVISGDKIN
GNCTGIKIYVSDDGKAHFSISNSAEDPFIAIHAESKL
>sp|P22998.1|AMY_STRVL
MARKTVAAALALVAGAAVAVTGNAPAQAVPPGEKDVTAVMFEWNFASVARECTDRLGPA
GYGYVQVSPPQEHLQGGQWWTSYQPVSYKIAGRLGDRTAFKNMIDTCHAAGVKVVADS
VINHMANGSGTGTGGTSFSKYDYPGLYSGSDMDDCRATISNYQDRANVQNCELVQLPDL
DTGEDHVRGKIAGYLNDLASLGVDGFRIDAAKHMPAADLANIKSRLTNPNVFWKLEAIH
GAGEAVSPSEYLGSGDVQEFRYARDLKRVLQGEKLSYLKNFGEAWGHMPSGQSGVFVDN
HDTERGGDTLSYKDGANYTLASVFMLAWPYGSPDVHSGYEWTDKDAGPPNNGQVNA
CYTDGWKCQHAWREISSMVAFRNTARGQAVTNWWDNGNNAIAFGRGSKAYVAINHETS
ALTRTFQTSLPAGSYCDVQSNTPVTVNSSGQFTATLAANTAVALHVNATGCGSTPTTPPTTP

PATSGASFNVTATTVVGQNIYVTGNRAELGNWAPASALKLDPATYPVWKLTVGLPAGTSF
EYKYIRKDAAGNVTWESGANRTATVPASGQLVLNDTFRS
>sp|P30270.1|AMY_STRGR
MARRLATASLAVLAAAATALTAPTPAAAAPPGAKDVTAVLFEWKFASVARACTDSLGPAG
YGYVQVSPPQEHIQGSQWWTSYQPVSYKIAGRLGDRAAFKSMVDTCHAAGVKVVADSV
INHMAAGSGTGTGGSAYQKYDYPGIWSGADMDDCRSEINDYGNRANVQNCELVGLADL
DTGEPYVRDRIAAYLNDLLLLGVDGFRIDAAKHMPAADLTAIKAKVGNGSTYWKQEAIH
GAGEAVQPSEYLGTGDVQEFRYARDLKRVFQNENLAHLKNFGEDWGYMASGKSAVFVD
NHDTERGGDTLNYKNGSAYTLAGVFMLAWPYGSPDVHSGYEFTDHDAGPPNGGTVNA
CYSDGWKCQHAWPELSSMVGLRNTASGQPVTNWWDNGGDQIAFGRGDKAYVAINHEG
SALNRTFQSGLPGGAYCDVQSGRSVTVGSDGTFTATVAAGTALALHTGARTCSGGGTGPG
TGQTSASFHVNATTAWGENIYVTGDQAALGNWDPARALKLDPAAYPVWKLDVPLAAGT
PFQYKYLRKDAAGKAVWESGANRTATVGTTGALTLNDTWRG
>sp|P56634.1|AMY_TENMO
QKDANFASGRNSIVHLFEWKWNDIADECERFLQPQGFGGVQISPPNEYLVADGRPWWER
YQPVSYIINTRSGDESAFTDMTRRCNDAGVRIYVDAVINHMTGMNGVGTSGSSADHDGM
NYPAVPYGSGDFHSPCEVNNYQDADNVRNCELVGLRDLNQGSDYVRGVLIDYMNHMID
LGVAGFRVDAAKHMSPGDLSVIFSGLKNLNTDYGFADGARPFIYQEVIDLGGEAISKNEYT
GFGCVLEFQFGVSLGNAFQGGNQLKNLANWGPEWGLLEGLDAVVFVDNHDNQRTGGSQ
ILTYKNPKPYKMAIAFMLAHPYGTTRIMSSFDFTDNDQGPPQDGSGNLISPGINDDNTCSN
GYVCEHRWRQVYGMVGFRNAVEGTQVENWWSNDDNQIAFSRGSQGFVAFTNGGDLNQ
NLNTGLPAGTYCDVISGELSGGSCTGKSVTVGDNGSADISLGSAEDDGVLAIHVNAKL
>sp|Q76L96|Q76L96_ASPAW
MRVSTSSLALSVSLFGKLALGLSAAEWRSQSIYFLLTDRFGRTDNSTTATCDTGDQIYCGG
SWQGIINHLDYIQGMGFTAIWISPITEQLPQDTSDGEAYHGYWQQKIYDVNSNFGTADDL
KSLSDALHARGMYLMVDVVPNHMGYAGNGNDVDYSVFDPFDSSSYFHPYCLITDWDNL
TMVQDCWEGDTIVSLPDLNTTETAVRTIWYDWVADLVSNYSVDGLRIDSVLEVEPDFFPG
YQEAAGVYCVGEVDNGNPALDCPYQDYLDGVLNYPIYWQLLYAFESSSGSISDLYNMIKS
VASDCSDPTLLGNFIENHDNPRFASYTSDYSQAKNVLSYIFLSDGIPIVYAGEEQHYSGGDV
PYNREATWLSGYDTSAELYTWIATTNAIRKLAISADSDYITYANDPIYTDSNTIAMRKG
TSGSQVITVLSNKGSSGSSYTLTLSGSGYTSGTELIEAYTCTSVTVDSNGDIPVPMASGLPR
VLLPAWVVDSSSSLWGGSTTTTTSSSTSTSTSKATSSSSTTTSSSCTATSTTLPITLEELVTTT
YGEEIYLSGSISQLGEWDTSDAVKLSADDYTSSNPEWYVTVSLPVGTTFEYKFIKVEEDGS
VTWESDPNREYTVPECGSGETVVDTWR
>sp|Alphaz|
MNRRTLGSLTGAAVLVTGLLTAPAPASATPAAPVPAAPAPGAVAAAEQNGGTIVHLFQWN
WDSVAAECEDFLGPNGFGGVQVSPPQEHVVIPSAEGGNHPWWQDYQPVSYQIDNTRRGT
AEEFEAMVATCRDNGVRIYADVIINHMTGPGSGTGSNGTEWEKYAYPDLFGDGSAAYGG
DDFGPCFETIDDWNDKWEVQNCELLELSNLNTASPHVRAQLTRYLNGLVEMGVGGFRVD
ASKHVAEADVEAIFGGLDAVPGFGGPPDVYHEVYGDQTVPYTAYTPYGRVTNFDYKNDF
AGKFAGGDIAGLVDMPDHGGLTADEAVVFVDNHDTQRYSPTLTYKDGDRYHLATAFMLA
HPYGTPVVMSSYDFGDNQTEGPPSTGDVEGNPAGWITEDTDCADSDWVCEHRDGTVAG
MAAFRNSTDGTGITQRAADGASRVAFDRGERGFAAFNAGGGTWNLTATTAMPDGAYENA
AGSGSATVSGGQVTVEVPAEGAFAIHVDGVCTDPAECDGDGDGDDGNTVQVSAHVETYF

GQEVYIVGETSALGSWNPASGARLSTDESTYPQWTGEAVIGPDDEWKLVKIDGSGAVEWE
SGANRVGPDTAPVWRN

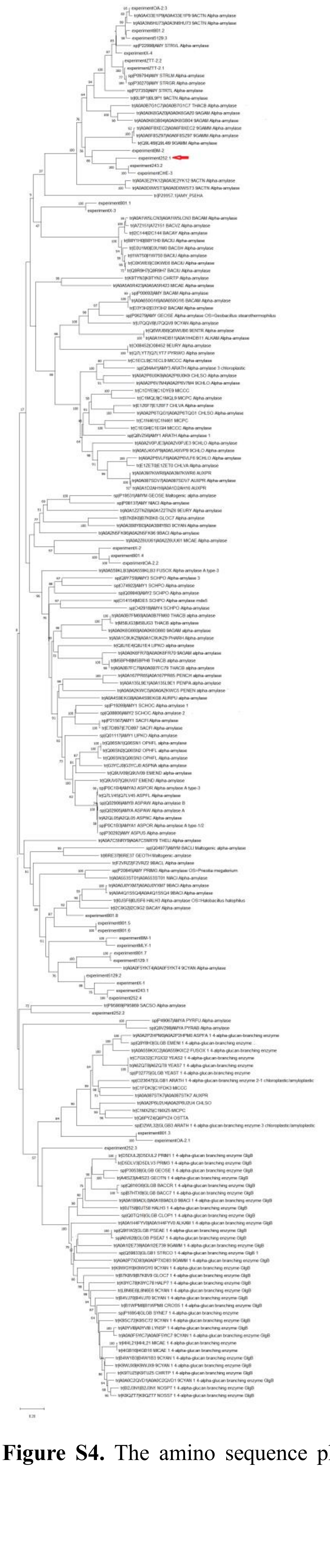

Figure S4. The amino sequence phylogenetic tree of strain HDN19-252. Bootstrap values are

shown on nodes in percentages of 500 replicates, when greater than 50%. Bar, 10 substitutions per 1000 nt.
